# Evaluating performance of existing computational models in predicting CD8+ T cell pathogenic epitopes and cancer neoantigens

**DOI:** 10.1093/bib/bbac141

**Published:** 2022-04-25

**Authors:** Paul R Buckley, Chloe H Lee, Ruichong Ma, Isaac Woodhouse, Jeongmin Woo, Vasily O Tsvetkov, Dmitrii S Shcherbinin, Agne Antanaviciute, Mikhail Shughay, Margarida Rei, Alison Simmons, Hashem Koohy

**Affiliations:** 1 MRC Human Immunology Unit, Medical Research Council (MRC) Human Immunology Unit, MRC Weatherall Institute of Molecular Medicine (WIMM), John Radcliffe Hospital, University of Oxford, Oxford, United Kingdom; 2 MRC WIMM Centre for Computational Biology, Medical Research Council (MRC) Weatherall Institute of Molecular Medicine, John Radcliffe Hospital, University of Oxford, Oxford, United Kingdom; 3 Department of Neurosurgery, Oxford University Hospitals NHS Foundation Trust, John Radcliffe Hospital, University of Oxford, Oxford, United Kingdom; 4 Nuffield Department of Surgical Sciences, University of Oxford, Oxford, United Kingdom; 5 Centre for Immuno-Oncology, Nuffield Department of Medicine, University of Oxford, Oxford, United Kingdom; 6 Chief Scientific Officer, ImmunoMind Inc, Berkeley, CA, USA; 7 Department of Genomics of Adaptive Immunity, Shemyakin-Ovchinnikov Institute of Bioorganic Chemistry, Moscow, 117997, Russia; 8 Institute of Translational Medicine, Pirogov Russian National Research Medical University, Moscow, 117997, Russia; 9 The Ludwig Institute for Cancer Research, Old Road Campus Research Building, University of Oxford, Oxford, United Kingdom; 10 Alan Turning Fellow, University of Oxford, Oxford, United Kingdom

**Keywords:** peptide immunogenicity, CD8 T-cell target, peptide presentation, T-cell response, cancer neoantigen, immunotherapy

## Abstract

T cell recognition of a cognate peptide–major histocompatibility complex (pMHC) presented on the surface of infected or malignant cells is of the utmost importance for mediating robust and long-term immune responses. Accurate predictions of cognate pMHC targets for T cell receptors would greatly facilitate identification of vaccine targets for both pathogenic diseases and personalized cancer immunotherapies. Predicting immunogenic peptides therefore has been at the center of intensive research for the past decades but has proven challenging. Although numerous models have been proposed, performance of these models has not been systematically evaluated and their success rate in predicting epitopes in the context of human pathology has not been measured and compared. In this study, we evaluated the performance of several publicly available models, in identifying immunogenic CD8+ T cell targets in the context of pathogens and cancers. We found that for predicting immunogenic peptides from an emerging virus such as severe acute respiratory syndrome coronavirus 2, none of the models perform substantially better than random or offer considerable improvement beyond HLA ligand prediction. We also observed suboptimal performance for predicting cancer neoantigens. Through investigation of potential factors associated with ill performance of models, we highlight several data- and model-associated issues. In particular, we observed that cross-HLA variation in the distribution of immunogenic and non-immunogenic peptides in the training data of the models seems to substantially confound the predictions. We additionally compared key parameters associated with immunogenicity between pathogenic peptides and cancer neoantigens and observed evidence for differences in the thresholds of binding affinity and stability, which suggested the need to modulate different features in identifying immunogenic pathogen versus cancer peptides. Overall, we demonstrate that accurate and reliable predictions of immunogenic CD8+ T cell targets remain unsolved; thus, we hope our work will guide users and model developers regarding potential pitfalls and unsettled questions in existing immunogenicity predictors.

## Introduction

The importance of being able to accurately predict CD8+ T cell targets, i.e. immunogenic peptides, has never been clearer than during the ongoing COVID-19 pandemic era. It is also central in devising personalized vaccines for various cancers.

An efficient antigen-specific CD8+ T cell response to exogenous pathogens or endogenous threats relies on tightly regulated processing and presentation of antigenic peptides by class I major histocompatibility complexes (MHCs) and subsequent recognition of the peptide–MHC (pMHC) by cognate CD8+ T cells [1, 2]. Therefore, immunogenic peptides encompass attributes associated to two sets of features known as peptide presentation and T cell receptor (TCR) recognition features [3]. Among these, features attributed to MHC presentation have been shown to be more prominent compared to those attributed to TCR recognition. Examples include heavily conserved anchor positions and enriched motifs associated with distinct HLA types [4, 5]. Indeed, recent cutting edge models in predicting MHC presentation have shown impressive performance, exemplified by the widely used NetMHCpan [6] and other recently published models [7].

The recognition features of peptides on the other hand are highly degenerated due to promiscuity of TCRs imposed by positive and negative selection to avoid immune blind spots [8]. In addition to peptides’ sequence-based recognition features, numerous other factors such as co-stimulation, proliferation and cytotoxicity underpin immunogenicity [1]. These factors collectively define the magnitude and shape of a T cell response and determine whether the response is elicited [9]. Furthermore, nuances in various experimental assays evaluating T cell responses can produce noisy data, while the lack of a ‘true negative’ pool of peptides augments this complexity. Indeed, a ‘negative’ T cell assay only means a peptide failed to elicit a T cell response in a given experiment, perhaps due to the absence of a cognate T cell. This does not necessarily mean that a peptide is objectively non-immunogenic. Taken together, the identification of ‘immunogenic’ peptides has proven to be more challenging than identification of peptides presented by MHC molecules. Further challenges—e.g. limited numbers of known TCR-pMHC pairs—in identifying T cell antigens have recently been reviewed by Joglekar and Li [10].

Despite these challenges, over the past decade, several models have been presented to predict immunogenic peptides, leveraging different correlates of immunogenicity with varying levels of success. As we recently detailed [8], a number of these studies have utilized sequence-based characteristics including amino acid features [11–15], similarity to viral peptides [16], sequence dissimilarity to self [3, 17], association between peptide immunogenicity and their biophysical properties such as their structural and energy features [18], as well as TCR recognition features [11, 19]. Recently, Wells *et al*. [3] comprehensively investigated a collection of parameters associated with neoantigen immunogenicity, grouped into (1) presentation features, e.g. binding affinity and stability, hydrophobicity and tumor abundance and (2) recognition features, e.g. agretopicity (the ratio of binding affinity between a mutated peptide and its wild-type counterpart), and foreignness (similarity of peptide of interest to previously characterized viral epitopes).

Despite the strong HLA restriction of peptide recognition by conventional T cells, with most immunogenicity models, presentation features have not been deconvoluted from more subtle T cell recognition features in this manner, which—due to the prominence of MHC features in sequence data—may lead to models primarily predicting HLA ligands rather than immunogenic peptides. A number of recent studies such as those by Wells *et al*. [3] and Schmidt *et al*. [19] have shed light on this issue by disentangling features associated with presentation versus those associated with T cell recognition [3, 19].

In addition to the emerging consensus that predicting peptide immunogenicity involves deconvoluting MHC presentation and T cell recognition features, evidence is suggesting that different features will be required to predict immunogenicity of pathogenic epitopes versus neoantigens, given fundamental differences in the mechanisms underpinning the respective T cell responses. Compared with pathogenic peptides, which are substantially different from the human proteome, neoantigens often exhibit only a single point mutation from the corresponding wild-type self-peptide [20]. This high sequence similarity between cancer neoantigens and self-peptides is likely subject to immune tolerance; thus, the focus has been placed on identifying features that permit neoepitopes to escape, which may be less applicable for pathogenic epitopes [21].

Indeed, Richman *et al*. [22] and Devlin *et al*. [23] have shown that dissimilarity to the self-proteome permits neoepitopes to escape from immune tolerance. However, we have recently shown that dissimilarity to self is limited in distinguishing immunogenic peptides from pathogens [21], which indicate differences in the features required to predict immunogenic peptides from pathogens versus cancer. Complicating matters further, existing models are primarily trained on pathogenic epitopes, some of which are used then to predict immunogenic neoantigens. Indeed, these important differences between pathogenic peptides and cancer neoantigens and their recognition by T cells indicate that separate features and training datasets would be required to reliably predict immunogenicity in these distinct settings.

Given this tapestry of complexity, it is unclear to what extent existing immunogenicity models can discriminate immunogenic peptides in human disease; although in mice, there is evidence that HLA ligand prediction alone may suffice in identifying such peptides. A recent study by Croft *et al*. [24] in vaccinia virus-infected C57BL/6 mice found that the majority (>80%) of presented peptides by H-2D^b^ or H-2D^k^ are immunogenic, implying that HLA ligand predictors could be accurately used to identify immunogenic vaccinia virus epitopes. This was evaluated in a recent study by Paul *et al*. [25], who benchmarked 17 models that are used primarily to predict antigen presentation. They showed each of these models can be used to predict immunogenic vaccinia virus peptides in mice at a relatively high level of confidence, albeit with some variation. They observed that NetMHCpan v4.0 was the best performing model. However, we have recently observed that the fraction of presented peptides in humans which are immunogenic may be more variable (2.4–69% across different studies) than observed by Croft *et al*. in mice [21]. It is unclear how much of this variation is a species-specific effect, or perhaps due to differences between experimental assays. Taken together, the extent to which immunogenicity models, as well as antigen presentation features, can predict peptide immunogenicity in the context of different human disease settings is an open question.

Numerous models over the past decade have been offered to predict peptide immunogenicity. These immunogenicity models have certainly contributed to our understanding of mechanisms underpinning T cell recognition in human disease; however, a systematic and unbiased evaluation of the performance of these models is not yet available and would provide a framework to guide identification of T cell targets in different immunological settings [3, 18, 20].

In this study, we evaluated and compared the performance of several publicly available models in predicting immunogenicity in human pathology settings. Given the crucial differences in—and features of—underlying mechanisms that invoke a T cell response, we illustrated the performance of these models in predicting immunogenic pathogenic epitopes and cancer neoantigens separately. In both settings, we found suboptimal model performance and considerable room for improvement. Furthermore, we explored several potential problems underpinning the suboptimal performance of some of these models and more generally the complexity of predicting peptide immunogenicity. We thus present a framework by which investigators can decide which immunogenicity classifier is more applicable to their data and research questions in the context of human disease.

## Results

### Evaluating model performance in predicting peptide immunogenicity

To perform an unbiased evaluation of existing models in predicting immunogenic peptides, we identified seven publicly available models (see Methods for criteria details), each of which aims to predict whether an MHC-presented peptide may invoke a T cell response (i.e. whether a peptide is *immunogenic*). These models are named as the *IEDB model* [14], *NetTepi* [13], *iPred* [12], *Repitope* [11], *PRIME* [19], *DeepImmuno* [15] and *Gao* [26] (Table 1, see Supplementary Text: Model Descriptions for detailed model overviews). Additionally, given the observation by Paul *et al*. [25] that NetMHCpan 4.0 most accurately identified immunogenic vaccinia virus T cell epitopes in mice, we included both eluted ligand (labelled *netMHCpan_EL*) and binding affinity (labelled *netMHCpan_BA*) outputs from NetMHCpan 4.0 to assess their accuracy in a human setting. We therefore evaluated the performance of nine models. We undertook the performance evaluation separately for pathogenic and cancer epitopes, due to inherent differences in these immunological settings.

**Table 1:** An overview of immunogenicity predictors that are evaluated in this study

Model	Model overview	Training data	Language	Input data	HLA restriction	Reported performance
IEDB Model Calis *et al.* [14]	The *IEDB model* captures sequence-based frequencies of specific amino acids to describe those which are more prevalent in immunogenic peptides compared with non-immunogenic peptides. The user is provided with a score between −1 and 1, to indicate likelihood of immunogenicity	Data were compiled from the IEDB and three murine studies Strict data curation process excluded humans as a host for non-immunogenic peptides. Nine-mers were then selected and a redundancy filtering process was performed to avoid oversampling Dataset after redundancy filtering is not publicly available	Python	Peptide(s) and HLA allele	Yes	ROC-AUC after 3-fold cross-validation: 0.65
NetTepi Trolle and Nielsen [13]	*NetTepi* was defined as the linear combination of binding affinity scores that were obtained by *NetMHCpan* algorithm, binding stability scores that are obtained by *NetMHCstab* and T cell propensity scores that are obtained similarly to the IEDB model	T cell propensity component employed training data as above for the IEDB model	Python	Peptides, HLA allele, peptide length (8–14)	Yes, 13 HLA-A and HLA-B alleles	Average AUC0.1 values range between 0.9305 and 0.9652
iPred Pogorelyy *et al.* [12]	*iPred* is a Multinomial Gaussian Process classifier, utilizing expectation-maximization. As input, *iPred* takes peptide sequence(s) and computes 10 Kidera Features averaged over all residues. The output of the model is a probability score reflecting the likelihood of T cell immunogenicity	Chowell dataset was used to compute Kidera factor vector sums for model training	R	Peptide sequence	No	ROC-AUC: ~0.80
Repitope Ogishi and Yotsuyanagi [11]	*Repitope* is a framework, which probes public TCRs to discriminate immunogenicity. *Repitope* computes physiochemical properties based on mimicking the thermodynamics between pMHC and public TCR interactions	Calis 2013 dataset, Chowell 2015 dataset, EPIMHC dataset, LANL HCV/HIV dataset, POPISK dataset, MHCBN dataset, TANTIGEN dataset. Please see following link for more details: https://github.com/masato-ogishi/Repitope	R or Python	Peptide sequences	No	ROC-AUC 0.76
PRIME Schmidt *et al*. [19]	*PRIME* predicts immunogenic epitopes through capturing and deconvoluting molecular properties of antigen-presentation and TCR recognition propensity. *PRIME* incorporates HLA binding predictions through MixMHCpred	Compiled from multiple studies. See Supplementary Table S1: https://pubmed.ncbi.nlm.nih.gov/33665637/	Python	Peptide sequences and a corresponding HLA allele for each peptide	Yes	ROC-AUC >0.7, PR-AUC >0.10 < 0.16.
DeepImmuno Li et al. [15]	DeepImmuno-CNN is a convolutional neural network approach, which predicts immunogenicity of pMHC complexes. Their immunogenicity score weights each pMHC complex based on the strength of available experimental evidence in the training dataset	Initial training and validation occurred using data from the IEDB. Please see: https://github.com/frankligy/DeepImmuno	Web portal or Python	Peptide sequence and corresponding HLA. Lengths 9/10 only	Yes	ROC-AUC 0.85 from cross-validation PR-AUC 0.81 from cross-validation
Gao Gao *et al*. [26]	A ‘physics-based’ learning model, aimed at predicting CTL epitopes. The model is trained and validated on peptides from HIV. The model defines a ‘CTL response metric’, which incorporates three terms that capture for a given peptide (1) HLA binding probability, (2) similarity to pathogenic peptides, (3) similarity to the human proteome	Peptide data from HIV patients	R	Peptide and HLA allele.	Yes	ROC-AUC 0.66–0.71

#### Evaluating model performance in predicting immunogenic pathogenic peptides

The ongoing efforts to understand T cell responses against severe acute respiratory syndrome coronavirus 2 (SARS-CoV-2) have provided the community with an unprecedented number of functionally evaluated SARS-CoV-2 peptides. A unique and valuable characteristic of this data megapool is that—with the exception of several peptides from other coronaviruses (e.g. MERS, SARS-CoV) which share homology with SARS-CoV-2 [27, 28]—many of these peptides have not been used in the training data of the models under investigation (Supplementary Fig. S1A available online at http://bib.oxfordjournals.org/) due to their recent identification. These peptides are therefore an ideal ‘test’ dataset for evaluating the performance of models in predicting immunogenic peptides from an emerging pathogen, enabling a benchmark of model performance in a realistic setting. Thus, we leveraged these data to shed light on the extent these models can be used to accurately identify immunogenic peptides upon the emergence of a novel pathogen.

**Figure 1:**
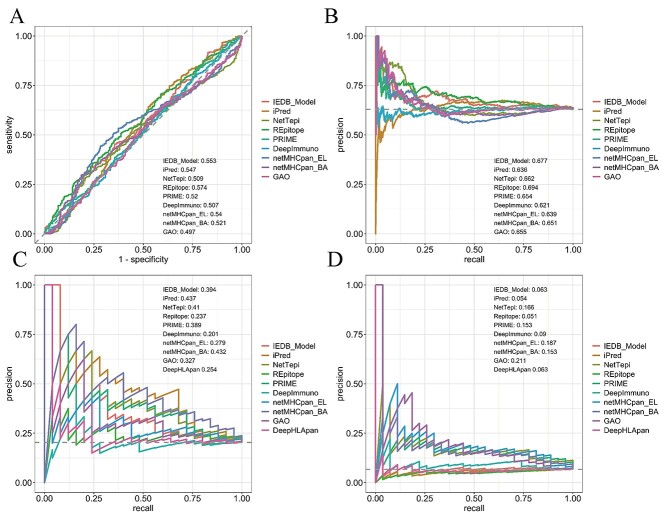
Models are not reliable in predicting epitopes for an emerging virus and exhibit room for improvement in predicting immunogenic cancer neoantigens. (**A**) ROC curves of models tested ‘as published’ against 858 SARS-CoV-2 peptides of lengths 9 and 10. (**B**) PR curves of models tested ‘as published’ against the SARS-CoV-2 peptides. (**C**) PR curves showing model performance against the GBM neoantigen dataset. (**D**) PR curves showing model performance against the TESLA neoantigen dataset.

To achieve this, we gathered all MHC class I SARS-CoV-2 peptides from the IEDB [29] with T cell response information and supplemented the dataset with further peptides from VIPR [30] (both databases were accessed 7 October 2021). Next, we applied two filters (see Methods for full details):

(i) Length filter: We limited ourselves only on 9- and 10-mers because (a) these lengths are the most common among CD8+ T cell targets and as a result the most prevalent lengths of class I peptides in our SARS-CoV-2 test data, (b) the sample sizes of peptides of lengths 8 (positive = 32, negative = 8), 11 (positive = 69, negative = 28), etc. were too low to draw robust conclusions and (c) these were the only lengths for which all nine models are applicable.
(ii) HLA filter: We limited ourselves on the 13 HLA alleles for which all models were applicable (see Methods).

Identical homologs from, e.g., MERS and SARS-CoV were retained (see Supplementary Fig. S1A available online at http://bib.oxfordjournals.org/); as upon the emergence of SARS-CoV-2, these conserved peptides may already have existed in model training data, therefore providing a more realistic testing scenario. Application of all filters left 858 SARS-CoV-2 peptides, of which ~63% were immunogenic and ~37% non-immunogenic. For added clarity, we assessed model performance using both receiver operating characteristic area under the curve (ROC-AUC), which is commonly used in machine-learning contexts, and precision–recall area under the curve (PR-AUC), which summarizes model precision and recall and more accurately represents the balance of classes within the testing dataset.

The ROC-AUCs obtained from this task ranged from 0.497 for *Gao* to 0.574 for *Repitope* (Fig. 1A), suggesting suboptimal performance for all models in identifying immunogenic epitopes from an emerging virus (Supplementary Fig. S1B, Supplementary Table S1 available online at http://bib.oxfordjournals.org/). PR-AUCs ranged from 0.621 for *DeepImmuno* to 0.694 for *Repitope* (Fig. 1B), which—given the proportion of immunogenic peptides in the dataset (~63%)—suggests that select models may perform marginally better than random. Indeed, a follow-up bootstrap analysis (see Methods) revealed that the majority of models did not perform substantially better than the baseline using these data; although, with varying levels of significance, *Repitope* (ROC-AUC *z*-score = 3.69, PR = AUC *z*-score = 3.87) and the *IEDB model* (ROC-AUC *z*-score = 2.54, PR-AUC *z*-score = 2.88) did perform better than random (Supplementary Fig. S1C–D, Supplementary Tables S2 and S3 available online at http://bib.oxfordjournals.org/).

Taken together, neither the assessed HLA ligand predictors nor models specifically designed to predict immunogenicity could reliably identify immunogenic peptides from an emerging pathogen. Thus, these data indicate that HLA ligand prediction is not sufficient to predict immunogenic epitopes from an emerging pathogen, suggesting that while presentation may be necessary it is not sufficient for T cell immunogenicity. Additionally, this analysis illustrates that current models, which have been designed to incorporate further features that describe T cell recognition of pMHCI complexes, do not appear to outperform peptide presentation predictions in this setting. These insights therefore reveal a gap for an immunogenicity predictor to help extract immunogenic peptides from the pool of presented pathogenic peptides.

### Evaluating model performance in predicting immunogenic cancer neoantigens

One key application for immunogenicity classifiers is to identify immunogenic cancer neoantigens that can activate CD8+ T cells for potential use as vaccine targets for personalized cancer immunotherapies [3, 31]. Identifying immunogenic neoantigens is a ‘needle in a haystack’ problem, where one aims to find extremely small numbers of ‘positives’ from substantially imbalanced datasets. Indeed, multiple studies have observed that among predicted candidate cancer neoantigen datasets, validated *immunogenic* neoantigens comprise ~6% [3, 32], suggesting high false positive rates among current identification pipelines. Nevertheless, in this scenario, the ability of models to accurately identify small numbers of positives is paramount.

For highly imbalanced classification, ROC-AUC can be misleading as this metric can underrepresent the minority class [33]. Thus, we diagnosed model performance in predicting immunogenic cancer neoantigens using PR-AUC [33]. We employed two independent cancer neoantigen datasets, both of which are intrinsically imbalanced: (1) our in-house glioblastoma (*GBM*) *dataset* and (2) a set of peptides gathered from the Tumor Neoantigen Selection Alliance (TESLA) consortium [3] (see Methods for details on both datasets). For this cancer neoantigen setting, we have additionally evaluated the model ‘*DeepHLApan*’ [34], which is an immunogenicity predictor designed to identify cancer neoantigens.

Our *GBM* dataset comprises peptides which bind HLA-A^*^02:01 from glioblastoma cancer patients. We excluded any peptides observed in any model’s training data. The resulting dataset comprised peptides of lengths 9 and 10 (*n* = 123), containing 25 (20%) confirmed immunogenic neoantigens. After testing these models against the *GBM* dataset, we observed suboptimal PR-AUCs, ranging from 0.20 for *DeepImmuno* to 0.437 for *iPred* (Fig. 1C, Supplementary Table S4 available online at http://bib.oxfordjournals.org/). With the exception of *DeepHLApan* which is hampered by false negatives, we observed a considerable number of false positives for each model (Supplementary Fig. S2A available online at http://bib.oxfordjournals.org/). Interestingly, *netMHCpan_BA, netMHCpan_EL* and *PRIME* identified the highest number (19, 18, 18 respectively) of the total 25 confirmed neoantigens (Supplementary Fig. S2A available online at http://bib.oxfordjournals.org/).

A bootstrap analysis revealed that despite suboptimal overall performance, the majority of models perform better than random (Supplementary Fig. S2B, Supplementary Table S5 available online at http://bib.oxfordjournals.org/). For example, *Z*-scores evaluating deviation of the true PR-AUCs from a distribution of those achieved by random predictions demonstrate that *netMHCpan_BA* (*z* = 5.2) possessed the most predictive power against this GBM dataset, followed by *iPred* (*z* = 5.03) and *NetTepi* (z = 4.38).

Next, we tested models against the publicly available ‘TESLA’ dataset. This dataset originally comprises cancer peptides among 13 class I alleles, of which we retained peptides experimentally tested against alleles for which all models are applicable, leaving peptides from seven HLAs. Additionally, we excluded any peptides observed in any model’s training data. These filters resulted in 27 (~6.7%) immunogenic and 372 (~93%) non-immunogenic peptides of lengths 9 and 10.

We again observed suboptimal PR-AUC scores for each model, ranging from 0.051 for *Repitope* to 0.211 for *Gao* (Fig. 1D; Supplementary Fig. S3A, Supplementary Table S6 available online at http://bib.oxfordjournals.org/)*.* After assessing predictive power as described previously, we observed that *Gao* (*z* = 6.65), *netMHCpan_EL* (*z* = 6.2), *NetTepi* (*z* = 4.88), *PRIME* (*z* = 4.55) and *netMHCpan_BA* (*z* = 4.53) each performed better than random (Supplementary Fig. S3B, Supplementary Table S7 available online at http://bib.oxfordjournals.org/). *Gao* utilizes dissimilarity to self and similarity to viral peptides to compute immunogenicity of a peptide, which are features identified by Wells *et al*. as important in discriminating immunogenic neoantigens in the context of the TESLA dataset, which may explain *Gao’s* higher performance here compared with the previous scenarios. *PRIME* identified the highest number of TESLA neoantigens (26/27) followed by *netMHCpan_EL* which identified (22/27). Here, we observed high numbers of false positives for all models, including *DeepHLApan* (Supplementary Fig. S3A available online at http://bib.oxfordjournals.org/).

Overall, these data highlight the complexity of predicting cancer neoantigens. Despite contribution of these models to fostering our understanding of T cell recognition of neoantigens, these analyses additionally illustrate there exists considerable room for improvement in accurately identifying immunogenic neoantigens. It is of note that for most models, their performance is hampered by a considerable false positive rate, which contributes to low precision. It is also important to note that although in some medical settings high false positive and low false negative rates are preferred, the ultimate aim of an immunogenicity classifier model is to achieve an optimal performance by balancing between false positive and false negative rates. Otherwise, models such as NetMHCpan capable of accurate prediction of peptide processing and presentation for CD8+ T cells would be sufficient to identify a target pool for further functional validation, as each *immunogenic* class I peptide should be presented. In this regard, the only model that exhibits a notable overall performance improvement compared with *NetMHCpan* is the *Gao* predictor, although this was only observed against the TESLA dataset. We also observed cross-data inconsistency in performance of these models in predicting cancer neoantigens. Taken together, these results suggest that an avenue for improving the performance of these models beyond what can be achieved by HLA ligand predictors is through improving the precision of these models to consistently identify the proportion of HLA ligands capable of invoking a T cell response.

In summary, we have illustrated suboptimal performance of existing models in predicting 9-mer and 10-mer targets for T cell responses for both emerging pathogens and cancers. In what follows we therefore explore a few potential features contributing to difficulties of predicting T cell target peptides.

### Exploring potential underlying issues with model performances

Here, we aim to shed light on issues and inconsistencies in model performances, which we hope can guide avenues of research for future immunogenicity predictors. First, given differences in model performances across the previously examined scenarios, we explore differences in underlying features associated with immunogenicity between pathogenic versus cancer settings, as well as potential reasons for contrasting performances within cancer settings. Secondly, due to HLA imbalances in training datasets and the low precision of the models, combined with their limited capacity to extend the performance achieved by HLA ligand predictor *NetMHCpan*, we explore the extent to which these models may primarily predict antigen presentation.

### Differences in discriminative features associated with immunogenicity for pathogenic versus cancer peptides

Despite broad application of these models in predicting peptide targets for CD8+ T cell responses in both cancer and infection settings, there is no systematic comparative analysis of features associated to cancer versus pathogenic peptide immunogenicity [21]. As discussed above in identifying immunogenic peptides in these two settings, we noticed inconsistent performances between cancer versus pathogenic scenarios as well as *within* cancer settings. For example, the *Gao* model was superior against one of two cancer datasets, but performed poorly against pathogenic epitopes. These inconsistencies suggest potential differences in parameters leading to pathogenic versus cancer neoantigen immunogenicity, which may translate into differences in the features required to discriminate immunogenicity in these two settings. We reasoned that such differences may contribute to the observed inconsistencies in model performances.

As mentioned, Richman *et al*. [22] and Devlin *et al*. [23] demonstrated that dissimilarity of a peptide to the self-proteome is associated with *neoantigen* immunogenicity. However, our recent work [21] has demonstrated that dissimilarity to self is limited in discriminating immunogenic pathogenic peptides. Indeed, Koncz *et al*. [35] recently reported that while a level of dissimilarity to the human proteome is critical to discriminate self and non-self, too dissimilar peptides were *less* likely to be immunogenic. The authors proposed that this could be explained through self-mediated positive selection, as T cells specific for peptides too dissimilar from human protein would not survive positive selection and would therefore be absent from the responding repertoire. Thus, while at lower levels dissimilarity to self may be able to assist in identifying, e.g. neoantigens with single amino acid variants from self, this feature may not exhibit a linear association with immunogenicity at higher levels of dissimilarity such as with pathogenic peptides.

Taken together, emerging evidence suggests that different features may be required to identify immunogenic peptides in pathogen versus cancer settings. Furthermore, given the wider availability of pathogenic compared with neoepitope datasets, immunogenicity predictors are often trained primarily on the former, which may further convolute predicting neoantigen immunogenicity.

For these reasons, we sought to investigate whether the observed variability in performances between pathogenic versus cancer settings may stem from differences in features attributed to immunogenicity. As discussed previously, Wells *et al*. [3] comprehensively interrogated features associated with neoantigen immunogenicity, defining those related to antigen-presentation or those related to T cell recognition. We therefore compared the capacity of these features previously associated with neoantigen immunogenicity, to discriminate immunogenic and non-immunogenic pathogenic peptides.

We first gathered class I associated pathogenic peptides from the IEDB (accessed 17 February 2022, ‘pathogenic’ dataset, see Methods for curation criteria), as well as the neoepitopes from Wells *et al*. [3] (TESLA ‘cancer’ dataset). For this analysis, we employed the entire TESLA dataset, comprising 608 peptides, of which ~6% were immunogenic and ~94% were non-immunogenic. The curated ‘pathogenic’ dataset consisted of 23 958 peptides in total, of which ~26% were immunogenic and ~74% were non-immunogenic. We then compared differences in the following presentation features: binding affinity and stability and the fraction of hydrophobic amino acids; the remaining features—agretopicity, foreignness and tumor abundance—are less applicable for pathogenic peptides.

**Figure 2:**
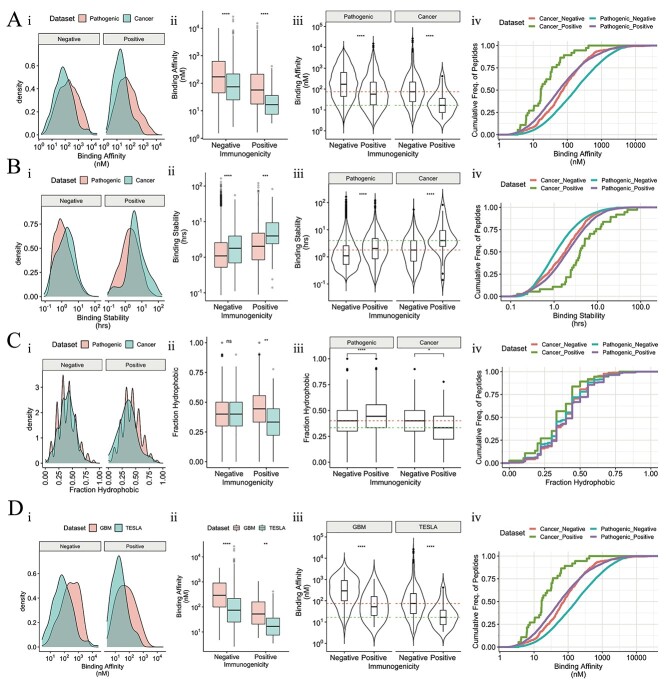
Differences in magnitude of discriminative antigen presentation features associated with immunogenicity for pathogenic versus cancer peptides: analysis was performed examining (**A**) binding affinities, (**B**) binding stabilities and (**C**) fraction of the peptide, which is hydrophobic (fraction hydrophobicity) between pathogenic and cancer peptides. (i) Density plots showing the distributions of binding affinities (A-i), binding stabilities (B-i) or fraction hydrophobicity (C-i) for immunogenic and non-immunogenic peptides within pathogenic versus cancer datasets. (ii) Boxplots comparing binding affinities (**A**-ii), binding stabilities (**B**-ii) or fraction hydrophobicity (**C**-ii) of immunogenic pathogen versus cancer peptides, as well as non-immunogenic pathogen versus cancer peptides. For fraction of hydrophobicity, the spikes for pathogenic peptides versus smoother distributions for cancer are due to differences in sample size. (iii) Violin or box plots comparing the binding affinities (**A**-iii), binding stabilities (**B**-iii) and fraction hydrophobicity (**C**-iii) of pathogenic immunogenic versus non-immunogenic peptides, as well as immunogenic versus non-immunogenic cancer peptides. Green and red dashed lines show the median of the respective measurement for the immunogenic and non-immunogenic cancer (TESLA) peptides, respectively. (iv) Line plots showing the empirical cumulative distributions of binding affinities (**A**-iv), binding stabilities (**B**-iv) and fraction of hydrophobicity (**C**-iv), grouped by whether the peptides are immunogenic or non-immunogenic for either cancer or pathogenic peptide datasets. (**D**-i) Density plots showing the distributions of binding affinities for immunogenic and non-immunogenic peptides between two independent cancer peptide datasets (GBM and TESLA). (**D**-ii) Boxplots comparing binding affinities of GBM versus TESLA peptides for both non-immunogenic and immunogenic peptides. (**D**-iii) Violin plots comparing the binding affinities of immunogenic and non-immunogenic peptides for GBM and TESLA cancer peptide datasets. Green and red dashed lines show the median of the binding affinities for the immunogenic and non-immunogenic cancer (TESLA) peptides respectively. (**D**-iv) Line plots showing empirical cumulative distributions of binding affinities for immunogenic versus non-immunogenic peptides for both cancer versus pathogenic datasets. Significance was assessed using Wilcoxon tests.

We observed that cancer peptides may exhibit stronger MHC binding affinities than their pathogenic counterparts (Fig. 2A-i). Indeed, for both non-immunogenic and immunogenic groups, cancer peptides possessed significantly stronger binding affinities compared with pathogenic peptides (Fig. 2A-ii). By comparing binding affinities of immunogenic versus non-immunogenic peptides grouped by pathogens or cancer, we observed that immunogenic pathogenic peptides had stronger binding affinities than non-immunogenic pathogen peptides, which was consistent with Wells’ observations for cancer peptides (2A-iii). Interestingly, the median binding affinity for *non-immunogenic* cancer peptides is similar to that of *immunogenic* pathogen peptides (2A-iii), in particular beyond ~100 nM (2A-iv), suggesting that thresholds learnt from training data to discriminate immunogenicity may need to be tailored to pathogen or cancer settings.

Different HLA alleles bind their ligands in different nM ranges [36]. Thus, the observed differences in nM binding affinities between pathogenic and cancer peptides could stem from different allele compositions of immunogenic and non-immunogenic peptides in these two datasets. To address this, we studied the NetMHCpan rank percentile values for these peptides (Supplementary Fig. S4A-i–iv available online at http://bib.oxfordjournals.org/) and observed the same patterns as shown in Fig. 2A, albeit more mildly. Importantly, we again observed similarities between the distributions of rank scores for *immunogenic* pathogen peptides and *non-immunogenic* cancer peptides.

For MHC binding stability, the trends were consistent with those of binding affinity. First, cancer peptides exhibited greater stability compared to their pathogenic counterparts (Fig. 2B-i and -ii). Similar to previous observations with binding affinity, we observed that while predicted binding stability can discriminate immunogenic and non-immunogenic pathogen peptides, the distributions of non-immunogenic cancer peptide stabilities are more comparable to those of *immunogenic* pathogenic peptide binding stabilities (Fig. 2B-iii and -iv). By studying the NetMHCStabPan rank percentile values, we again observed the same patterns as with stability in hours shown in Fig. 2B, albeit more mildly (Supplementary Fig. S4B-i–iv available online at http://bib.oxfordjournals.org/).

To minimize cross-HLA variation, one may need to take a per-HLA approach to compare discriminative features of peptide immunogenicity between pathogenic and cancer peptides. However, low sample size in the TESLA dataset (247 non-immunogenic versus only 12 immunogenic for the most common allele HLA-A*02:01) would not permit sound conclusions to be drawn. Notwithstanding, we were able to compare the capacity of these features to discriminate *pathogenic* immunogenic and non-immunogenic peptides for five common HLAs. With the exception of HLA-C*07:02 (possibly due to limited numbers of peptides for this HLA), we observed that immunogenic peptides had stronger binding affinities (Supplementary Fig. S4C available online at http://bib.oxfordjournals.org/) and more stable binding (Supplementary Fig. S4D available online at http://bib.oxfordjournals.org/) than non-immunogenic ones.

Together, these data provided evidence that while these features can discriminate immunogenic peptides, employment of the same thresholds or weights for MHC binding affinity or stability in both pathogen and cancer settings is likely to be ineffective. These observations of stronger binding affinities and more stable binding for cancer peptides may be due to facets of neoantigen identification pipelines, where one screens for high antigen presentation metrics to increase the likelihood of selecting better neoantigen candidates. Additionally, the effect of cross-HLA variation cannot be ruled out. This pre-selection for high presentation metrics may in turn contribute to skews in the datasets available for inference of MHC binding thresholds in discriminating immunogenic neoantigens.

As it comes to the ‘fraction of hydrophobicity’, we found it interesting that immunogenic pathogen peptides were more hydrophobic than their cancer counterparts (Fig. 2C-i and -ii). This feature can indeed discriminate immunogenic and non-immunogenic peptides in both pathogenic and cancer settings, although for pathogens there is a stronger trend, and in the reverse direction compared with cancer peptides (Fig. 2C-iii and -iv).

Next, as certain HLAs possess preferences for hydrophobic residues, we explored the capacity of this feature to discriminate immunogenic pathogen peptides across a set of common HLAs. Here, we observed variability in discriminating immunogenic peptides, suggesting this feature may need to be considered in an HLA-specific manner (Supplementary Fig. S4E available online at http://bib.oxfordjournals.org/). While these data suggest that there is perhaps more weight for this feature in identifying immunogenic pathogen peptides versus cancer—albeit in the reverse direction—further work is required to determine whether this is a biological characteristic or a technical artefact. Of note, we did not observe any significant differences of ‘fraction of hydrophobicity’ at TCR contact positions [35] for immunogenic and non-immunogenic peptides among cancer and pathogenic peptides (S5F), suggesting that the capacity of this feature to discriminate immunogenic peptides is primarily derived from anchor positions.

Inconsistencies in performances were also observed after testing the models against two independent cancer datasets. We therefore sought to examine whether immunogenicity parameters are likely to be linked to these inconsistencies. Here, we observed that binding affinity nM (Fig. 2D-i–iv) and rank score (Supplementary Fig. S5A-i and -ii available online at http://bib.oxfordjournals.org/), binding stability in hours (Supplementary Fig. S5B-i and -ii available online at http://bib.oxfordjournals.org/) and rank score (Supplementary Fig. S5B-iii and -iv available online at http://bib.oxfordjournals.org/), as well as fraction of hydrophobicity (Supplementary Fig. S5C-i and -ii available online at http://bib.oxfordjournals.org/) can discriminate immunogenicity for both cancer datasets (GBM and TESLA). Consistent with the results comparing pathogens versus cancer, these data indicate that the thresholds required to discriminate immunogenicity are again likely to be different between the datasets. We were unable to explore the ‘recognition’ features associated with neoantigen immunogenicity as proposed by Wells, as our GBM dataset does not have tumor abundance information, which is a pre-requisite for the application of Wells’ recognition features. This task suggests that a fixed set of parameters may not be universal to all cancer datasets and that user input regarding the appropriate set of parameters, as well as interpretation of results, will be crucial in identifying immunogenic cancer neoantigens.

Overall, we have observed evidence for differences in the thresholds required for features to discriminate peptide immunogenicity in pathogens versus cancer. Our observations indicate that a separate examination of presentation features and their weights in association with pathogen or cancer peptide immunogenicity is warranted. Furthermore, immunogenicity models are often trained primarily on pathogenic peptides. Therefore, as the distributions of binding affinities and/or stabilities for *immunogenic* pathogen peptides and *non-immunogenic* cancer peptides are comparable, this may in-part contribute to the substantial numbers of false positives generated by the models in identifying immunogenic cancer neoantigens, as well as inconsistencies in model performances between these immunological settings.

#### The effects of cross-HLA variation on predicting peptide immunogenicity

By exploring per HLA distributions of immunogenic and non-immunogenic peptides in the training data of these immunogenicity models, we observed substantial cross-HLA variation. Examples of these can be observed in *Repitope* (Supplementary Fig. S6A available online at http://bib.oxfordjournals.org/), the *IEDB model* (Supplementary Fig. S6B and C available online at http://bib.oxfordjournals.org/) and *iPred* (Supplementary Fig. S6D available online at http://bib.oxfordjournals.org/), where per HLA differences in numbers of positive (immunogenic) and negative (non-immunogenic) peptides are apparent. In fact, for *iPred*, we observed that immunogenic peptides dominate the HLA-A supertype, whereas non-immunogenic peptides dominate HLA-B presented peptides in the training data (Supplementary Fig. S6D available online at http://bib.oxfordjournals.org/). As a result, HLA-A presenting peptides are more likely to receive a higher immunogenicity score from this model, regardless of their true immunogenicity status (Supplementary Fig. S6E available online at http://bib.oxfordjournals.org/).

We therefore sought to evaluate whether cross-HLA imbalances in immunogenic and non-immunogenic peptides may have contributed to suboptimal performances of some of the models. We hypothesized that without explicitly deconvoluting antigen-presentation features from TCR recognition propensity, training these models on HLA imbalanced datasets may lead to skewed predictions associated to HLA. This may in turn lead to predictions primarily of antigen presentation—due to more prominent MHC features [4, 7]—rather than reflecting both peptide presentation and subsequent TCR recognition. We therefore aimed to explore whether the immunogenicity models (i.e. excluding HLA ligand predictors) exhibit skewed prediction scores toward dominant HLAs and whether models thus predict prominent HLA types.

**Figure 3:**
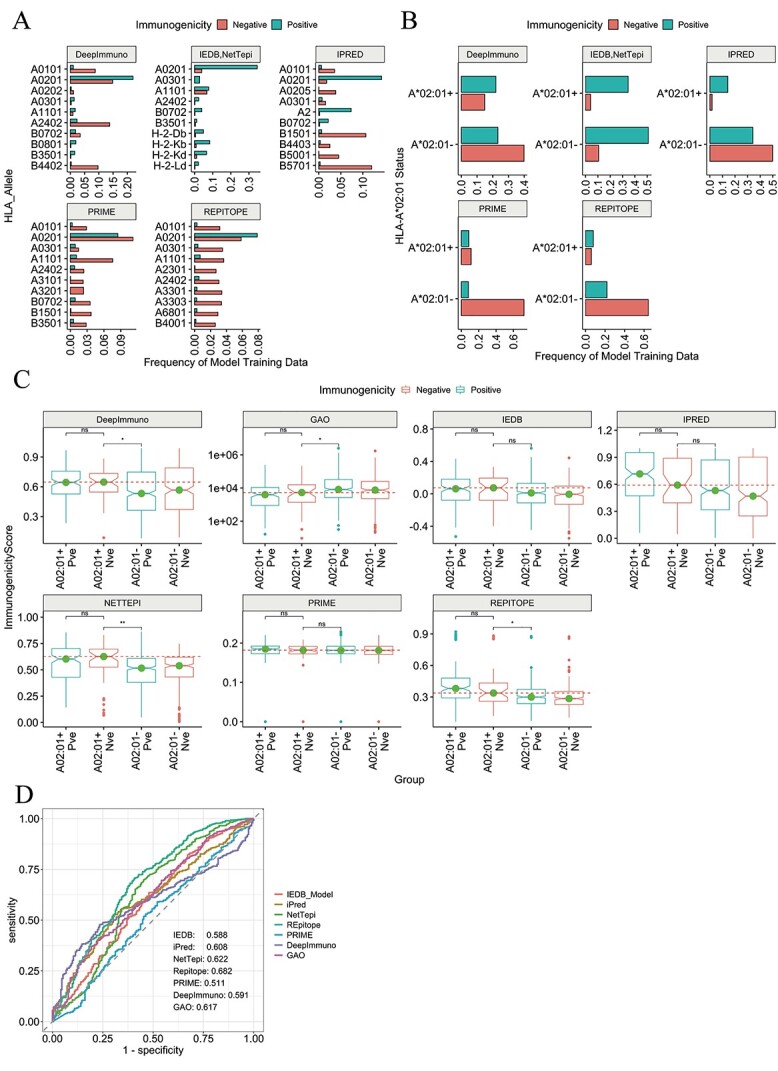
Effects of cross-HLA variation on predicting peptide immunogenicity. (**A**) Bar plots showing the distribution of immunogenic and non-immunogenic peptides for the top HLA alleles in each model’s training dataset. Gao’s training data are not included as the immunogenicity status is continuous. (**B**) Bar plots showing the distribution of immunogenic and non-immunogenic peptides for whether they bind HLA-A02:01 (labelled A*02:01+) or another allele (A*02:01-). (**C**) Notched boxplots showing subtle differences between the immunogenicity scores created by each model, based on whether the peptide binds HLA-A*02:01 (A02:01+) or another allele (A02:01-), and whether the peptide is immunogenic (Pve, labelled blue) or non-immunogenic (Nve, labelled red). For ‘Gao’, the *y*-axis is log10 scaled. (**D**) ROC-AUCs of the models after taking scores produced to predict immunogenicity for SARS-CoV-2 peptides, but instead asked to predict HLA-A02:01 status. Significance was assessed using Wilcoxon tests.

We first examined the distributions of the 10 (for consistency among models) most dominant HLAs in each model’s training data. We observed large skews toward and among HLA-A*02:01 for all models (Fig. 3A). Indeed, by grouping peptides in each model’s training data by whether they bind HLA-A*02:01 (hereby A*02:01+) or not (A*02:01-), we observed considerable differences in the frequency of immunogenicity status per model training data (Fig. 3B). We observed that with the exception of *IEDB* and *NetTepi*, non-immunogenic peptides dominate the A*02:01- group, meaning that non-immunogenic peptides are primarily presented by HLAs other than HLA-A*02:01 in model training data. It is plausible that this skewed distribution of prominent HLA features associated to immunogenicity status may lead to predictions skewed by HLA type.

We therefore hypothesized that these models may recognize prominent sequence features among HLA-A^*^02:01-binding peptides in test datasets, leading to inappropriately skewed model predictions. Therefore, we gathered the immunogenicity prediction scores generated by each model after evaluating their performance against our SARS-CoV-2 9- and 10-mer peptide dataset and grouped scores by whether the peptide is immunogenic or not, and whether it binds HLA-A*02:01 or not. First, for many examined models, we did not observe significant differences in immunogenicity scores between immunogenic and non-immunogenic peptides which bind HLA-A^*^02:01 (Fig. 3C) (labelled A02:01+ Pve and A02:01+ Nve, respectively). Furthermore, we found that *Repitope* (Cohen’s *d* = 0.454), *DeepImmuno* (Cohen’s *d* = 0.347), *NetTepi* (Cohen’s *d* = 0.338) and to some extent *iPred* (Cohen’s *d* = 0.206) predict *non-immunogenic* peptides that bind HLA-A^*^02:01 with higher scores than *immunogenic* peptides that bind remaining alleles; where on the contrary *immunogenic* peptides are expected to be predicted with higher immunogenicity scores. This analysis suggests that these models recognize dominant skewed HLA features associated with immunogenicity imbalances, which inappropriately skews model prediction scores, regardless of the true immunogenicity status of the peptide.

We next sought to analyze the extent to which these models detect peptide features associated with antigen presentation as opposed to T cell recognition. Here, we utilized prediction scores generated during the SARS-CoV-2 pathogenic epitopes analysis to instead predict whether a peptide engages HLA-A*02:01 or not (Fig. 3D). Remarkably, we observed higher performance here for all models, compared with when tasked with predicting SARS-CoV-2 peptide *immunogenicity* (Fig. 3D versus Fig. 1A), implying that for peptides from an emerging virus, all assessed immunogenicity predictors more capably predict dominant HLA features than T cell immunogenicity.

Overall, our data suggest that cross-HLA variation in the distribution of positive and negative peptides in model training data is highly likely to affect the peptide immunogenicity prediction, in a sense that some of these models in their existing settings predict HLA type more accurately than peptide immunogenicity. This insight suggests that suboptimal performance of these models is partly due to data limitations. Furthermore, our data suggest that future training datasets composed of more balanced immunogenic and non-immunogenic peptides for various HLA (or at least the common class I HLAs) are required for more accurate immunogenicity predictions. However, our work indicates that in the absence of such comprehensive datasets, the modelling strategies should consider how information extracted from HLA imbalances in training data affect model predictions and that future immunogenicity models should carefully model HLA restriction of T cell recognition.

## Discussion

In this study, we have demonstrated that despite great efforts, there is room for improvement to achieve accurate and reliable predictions of CD8+ T cell targets for an emerging virus such as SARS-CoV-2, or for a tumor of interest for the purpose of developing personalized (or stratified) treatments. We have additionally highlighted several issues underpinning suboptimal performances of these models with the hope of (a) making potential users aware of these issues and guiding them towards strategies for use of an appropriate model for their tasks, and (b) pointing future model developers to standing issues for further advancements and improvements.

For predicting CD8+ T cell targets for an emerging virus, these models do not seem to offer much improvement beyond MHC binding or presentation scores generated by models which predict HLA binding status such as *NetMHCpan*, i.e. they may not offer much help extracting peptides that will trigger T cell response from the pool of presented peptides. For predicting immunogenic cancer neoantigens, we again illustrated that the assessed models only marginally outperform *NetMHCpan*. *PRIME* and *NetTepi* were able to identify a high proportion of immunogenic neoantigens (high Recall), albeit at the expense of many false positives (low precision). Indeed, in most cases in this setting, the models produced many false positives. As immunogenic class I peptides must be presented by MHC, HLA ligand prediction with 100% accuracy could theoretically include 100% of immunogenic peptides (high recall), albeit with a high proportion of false positives (low precision) from those presented peptides which are not immunogenic. This concept is consistent with most observed model performances, and perhaps may in-part explain low precision observed with these models, as multiple predictors incorporate—and barely extend performance of—antigen presentation predictions.

Consensus is indeed emerging that presentation of a mutated peptide is insufficient for neoantigen immunogenicity [3, 20, 32]. Our findings are consistent with this concept and indicate that additional features are required to consistently discriminate HLA ligands that can or cannot invoke T cell responses. Recently, the work of Wells *et al*. has provided a paradigm for such an approach. Consistent with Wells *et al*., our work suggests that parameters associated with neoantigen immunogenicity may require calibration for individual use cases. Further factors, e.g. differences between cancers, technical variations between experiments, and inherent human variation, are likely to compound this complexity.

Several recent studies have investigated some additional parameters associated with peptide immunogenicity that were not covered in the models that we have evaluated [3, 18, 20]. Riley *et al*. [18] presented a structure-based approach, reporting increased performance against other models including the *IEDB model* and *NetTepi*. Despite this success, the authors suggest improvements to their model are necessary before wide adoption of their methodology. To our knowledge, their predictor is not yet publicly available, thus we were not able to evaluate its performance in the present study. Capietto *et al*. [20] recently supplied a framework for how mutation position contributes to neoantigen immunogenicity and proposed that the suboptimal landscape of neoantigen prediction stems from a limited number of available tools which capture a variety of features associated with neoantigen immunogenicity. Indeed, future work should seek to examine the full spectrum of available parameters associated with neoantigen immunogenicity.

**Table 2:** An overview of suggested applications for select immunogenicity predictors

Immunogenicity scenario	Recommended model(s)	Notes/justification
Predicting epitopes from an emerging pathogen or pathogenic epitopes in general	NetMHCpan + Repitope	Each model performed poorly in this setting (Fig. 1A); however, Repitope exhibited the most predictive capacity. NetMHCpan should be used to first predict HLA binders, followed perhaps by Repitope to suggest immunogenic peptides. Users should consider potential issues with regard to cross-HLA variation that may arise from Repitope
Identifying immunogenic neoantigens	1) PRIME, NetTepi, NetMHCpan, Gao.	While suboptimal performances were observed for each model, PRIME identified the highest number of immunogenic peptides albeit with limits in precision. NetTepi, NetMHCpan and Gao also showed potential to identify immunogenic peptides. The desired precision versus recall for a given research question should dictate which model is used If all features are available, the TESLA algorithm has shown potential in other work

By investigating several potential factors contributing to suboptimal performance of these models in the identification of immunogenic viral or cancer peptides, our work pointed towards both data- and model-associated issues. Data-associated issues include (i) small sample numbers especially for uncommon HLAs, an issue which is compounded for neoantigens, (ii) cross-HLA imbalances of positive and negative peptides and (iii) lack of clarity on true non-immunogenic peptides available in training datasets. Furthermore, the immunogenicity of a peptide is determined using different functional T cell response assays, which adds further noise to the data. With regard to cross-HLA imbalances, our findings suggest that skews in the distributions of immunogenic and non-immunogenic peptides per HLA in model training data may introduce bias into predictions. This observation is supported by Bassani-Sternberg *et al*. [7], where they showed that sequence similarity (i.e. HLA-I binding motifs) could effectively cluster peptides by respective HLA allele binding.

Model-associated issues suggested by our work include the use of universal parameter values and features for the identification of both viral and cancer antigens, training models on pathogenic peptides which are then used for prediction of cancer neoantigens and vice versa and limited consideration of HLA-restriction criteria of T cell recognition. We speculate that collectively, such issues may contribute to the limited precision of these models in identifying immunogenic neoantigens.

Although in this study we sought to provide a general and an unbiased evaluation of the performances of existing immunogenicity models, there exist several limitations to our approach that are worth highlighting. First, we had to limit ourselves to specific HLAs and on peptide lengths (9 and 10) for which all models are applicable. More data from other HLAs and peptide lengths would be required for generalization of our observations. Second, despite some potential benefits in re-training these models to further assess their reliability in different research settings, we chose to employ the models as trained by their authors. Key justifications for this are as follows: (a) the aim to provide a fair comparison across models; (b) in most cases the end users would employ a model ‘as published’ and would not re-train them themselves. Additionally, retraining models may require recalibration of their parameters given new training datasets.

Third, for evaluating performance of models in identifying immunogenic peptides for emerging pathogens, we performed this only—as a proof of concept—with a SARS-CoV-2 dataset that has not been used in training datasets of the models. Therefore, cross-pathogen variation in immunogenicity prediction is likely and highly important, but it is beyond the scope of this study. Finally, for our work regarding how HLA imbalances affect model predictions, we could only feasibly assess HLA-A*02:01 versus remaining alleles, rather than HLA-A*02:01 versus specific HLAs. More data from other HLAs will permit HLA-specific analyses.

The work of Croft *et al*. [24] indicated that vaccinia virus peptides presented by mice MHC molecules are highly translated into T cell recognition and response. It is therefore not surprising to see that in mice HLA ligand predictors, e.g., NetMHCpan 4.0 may accurately identify immunogenic epitopes [25]. However, as we have observed in the present study and also previously reported [21], in humans HLA presentation does not seem to be sufficient for T cell recognition. Therefore, additional features of peptide immunogenicity are required to assist extracting T cell targets from the pool of presented peptides in humans.

We envision that the insights provided in this study can assist end users to make evidence-based decisions for which model and parameters to use with their data and research questions.

Our work suggests that presentation features binding affinity, peptide stability and fraction of hydrophobicity are all associated with peptide immunogenicity for both cancer neoantigens and viral peptides albeit with different parameter thresholds. While recognition features foreignness and agretopicity are associated with neoantigen immunogenicity, they are likely to be less applicable to viral peptides. Our work additionally suggests that for both cancer and pathogenic CD8+ T cell target identification, the first reliable filter would be their presentation status, which is predicted by tools such as NetMHCpan. For pathogenic peptides, this could then be followed by additional filters, e.g. *Repitope* (Table 2). For cancer neoepitopes, *PRIME*—which incorporates presentation predictions—could be employed although users should expect high levels of false positives.

## Conclusions and future directions

Our work highlights a need for development of more accurate models for prediction of CD8+ T cell targets from emerging pathogens such as SARS-CoV-2 as well as cancer neoantigens. Such accurate and reliable models would assist with several burning challenges, e.g. facilitate use of personalized immunotherapies or permit investigation of the effect of mutations in CD8+ T cell targets on immunogenicity, to list a couple.

Our work suggests that suboptimal performance primarily stems from data-associated issues, although there also exist model-associated issues. Several challenges should thus be addressed. Further experimental datasets incorporating more diverse HLA restriction would help to reduce potential bias in the data. Deconvoluting information extracted from anchor positions versus TCR contact position, as opposed to modelling only full-length peptides appears to be an attractive approach to carefully incorporate HLA restriction, such as with work by Wells *et al*. [3] and Schmidt *et al*. [19]. Considering the lack of true negative data, positive unlabeled learning (where only positive observations are labelled) or other semi-supervised learning models to make use of both labelled and unlabeled data, e.g. generative variational autoencoder models seem appealing options.

Comprehensive identification of key features associated with peptide immunogenicity remains incompletely addressed for both cancers and pathogens. Separate models or approaches are likely required to predict viral antigens versus immunogenic cancer neoantigens. Future high throughput sequencing data with pMHCs coupled with their cognate TCRs are expected to boost the accuracy of future models.

The observation that existing models are not generalizable to predict immunogenic peptides from emerging viruses might be of utmost immunological importance. The underlying reasons remain to be fully addressed.

We envision that addressing such concerns would create vast potential for highly accurate immunogenicity predictors, which could augment the efficiency of medical research and managing pandemic disease. We hope this study will assist future model developers in addressing issues highlighted here and also to guide design of future experiments to provide the required data.

## Methods

### Models selected for analysis

First, we gathered all publicly available models and excluded those which we were unable to perform comparative assessment. After exclusion (see Supplementary Table S8 available online at http://bib.oxfordjournals.org/), we were left with seven models: the *IEDB model*, *NetTepi iPred*, *Repitope*, *PRIME*, *DeepImmuno* and *Gao*. NetMHCpan 4.0 eluted ligand and binding affinity scores were additionally evaluated. The study reporting ‘PRIME’ focuses on predicting immunogenic neoantigens; however, the model is presented also as a predictor of pathogenic epitopes. All models with the exception of ‘*DeepImmuno*’ were downloaded and ran locally. We were unable to run *DeepImmuno* locally for unknown technical issues; therefore, we instead used the webserver at https://deepimmuno.research.cchmc.org/

### Data analysis

All analyses were performed in R 4.0.3. All visualizations (excluding ROC and PR curves) were produced using either the *ggpubr* or *ggplot2* packages. ROC curves were produced using the *pROC* package and PR curves were produced using the *yardstick* package. Confusion matrices and assessment metrics were computed using the *caret* package.

### Definition of immunogenic peptides

In the current study, we refer to immunogenic peptides as those which possess a ‘positive’ label in data repositories such as the IEDB. This label refers to peptides where a T cell response has been observed by, e.g., an IFNγ ELISpot assay, although other techniques are commonly used.

### Model training data acquisition

We obtained the IEDB model training data from the supporting information of Calis *et al*. 2013. This version of the data is prior to their redundancy filtering step, and to our knowledge, the final training data are not publicly available. The ‘Chowell’ dataset used to train iPred was obtained from the github repository hosting the classifier: https://github.com/antigenomics/ipred/tree/master/classifier

The *MHCI_Human* training data for the human class I Repitope model were obtained from the R package, hosted at https://github.com/masato-ogishi/Repitope

Training data for ‘PRIME’ were downloaded from the supplementary of the original publication [19]. Training data for ‘DeepImmuno’ were downloaded from https://github.com/frankligy/DeepImmuno#deepimmuno-cnn

### Performance evaluation

All models were executed with default settings. For models where HLA restriction is considered, the corresponding HLA was provided to the model for each peptide of interest. For *NetTepi*, the length of the peptide is also provided at the command line.

For each model, ROC curves were built using the *pROC* package and PR curves built using *yardstick*. To compute confusion matrices, binary classifications must be generated. Thus, from ROC curves, optimal threshold values for binary classification (Positive or Negative) were generated using the Youden index. The Youden index uses ROC curves to compute a threshold value which maximizes the equation (1-sensitivity+specificity). For each model, the individual computed threshold value was used to classify prediction scores into ‘Positive’ or ‘Negative’ sequences and compiled in an additional ‘*ImmunogenicityPrediction*’ column. For each model, confusion matrices were generated using the *confusionMatrix* function in the *caret* R package.

In each experiment—with the exception of SARS-CoV homologs in the SARS-CoV-2 experiment—peptides observed in model training data were excluded from performance evaluations. Model performance was evaluated through a combination of ROC-AUC, PR-AUC, precision and recall. Other metrics such as *F*1 score and Balanced Accuracy were also calculated. ROC-AUC curves show the performance of a model by perturbing thresholding and visualizing the true positive rate (fraction of true positives/all true positives) against the false positive rate (fraction of false positives/all true negatives). Curve information is summarized using the AUC. Given a balanced dataset for binary classification (50% each classification), a random, unskilled model will have a ROC-AUC of 0.5, reflecting only the balance in the dataset. In contrast, a perfect model would have a ROC-AUC of 1.0. In a similar fashion, PR curve is a visualization of model precision and recall (equations are described below) after perturbing thresholds. A perfect model would have a PR-AUC of 1.0, and a no skill classifier would reflect the balance in the data, i.e. using the above example, PR-AUC would be 0.5.

$$ \mathrm{Precision}=\frac{\mathrm{tp}}{\mathrm{tp}+\mathrm{fp}} $$

$$ \mathrm{Recall}=\frac{\mathrm{tp}}{\mathrm{tp}+\mathrm{fn}} $$

where `tp' stands for `true positives', `fp' stands for `false positives' and `fn' stands for `false negatives'.

### Evaluating model performance against SARS-CoV-2 peptides

The SARS-CoV-2 data were downloaded from the IEDB and VIPR [30] (both accessed 7 October 2021). Data were first filtered for class I binding peptides only (i.e. HLA allele information containing phrases *HLA-A*, *HLA-B* or *HLA-C*). We excluded 20 pMHC that possessed only a single immunogenic observation but two or more non-immunogenic observations, which suggests that the positive assay could not be replicated. Otherwise, if contradictory pMHC observations (i.e. peptide *A*, HLA *y*, immunogenicity both Positive and Negative in different experiments) were observed, the observation was assumed ‘positive’. NetTepi is only applicable for 13 HLA alleles (see below); therefore, we only retained peptides assessed in the context of these alleles. We then filtered on 9- and 10-mers, as *DeepImmuno* is only able to predict immunogenicity for these lengths. Forty-nine SARS-CoV-2 peptides were observed in the collective model training data. After inspection, these peptides were from other coronaviruses. Given our aim to emulate a scenario with an emerging pathogen (where some peptides may have homology to other pathogens, but most are ‘unseen’), these were retained.

Each model was executed as published and prediction scores were generated for each peptide. ROC-AUC, PR-AUC, assessment metrics and confusion matrices were generated as described previously.

Alleles for which NetTepi is applicable: HLA-A*02:01, HLA-B*58:01, HLA-B*15:01, HLA-B*35:01, HLA-B*07:02, HLA-A*01:01, HLA-A*03:01, HLA-A*11:01, HLA-A*24:02, HLA-A*26:01, HLA-B*27:05, HLA-B*39:01, HLA-B*40:01.

### Bootstrap analysis to assess model predictive power

To assess predictive capacity of models against a test dataset, we first gathered the prediction scores generated by each model against the test dataset. These scores were not altered; however, we randomly shuffled the immunogenicity label for each peptide. After each shuffling, we calculated PR-AUCs between the original model prediction scores and the newly shuffled immunogenicity labels. This process was repeated 1000 times, resulting in a distribution of ‘shuffled PR-AUCs’, reflecting performance of a distribution of random, unskilled models. We then compared the true ‘benchmarked PR-AUCs’ against this distribution of ‘shuffled PR-AUCs’, to provide a representation of how superior the model performance observed during benchmarking was to a distribution of unskilled models. *Z*-scores were calculated by subtracting the mean of the ‘shuffled PR-AUCs’ from the benchmarked PR-AUCs and then by dividing the standard deviation of the ‘shuffled PR-AUCs’.

### Generating the ‘GBM’ dataset

These peptides were predicted using an in-house neoantigen identification pipeline and subsequently functionally validated. The neoantigen prediction workflow consisted of calling somatic variants from patient whole-exome sequencing data, which were used as inputs to an in-house version of MuPeXI [37], which we named `TUNAPASTA v0.5'. TUNAPASTA v0.5 was developed to accept the required data format but changes were not made to MuPeXI's ranking approach. Prior to functional validation, these peptides were filtered to be of TUNAPASTA neoantigen priority score >50. Selected peptides were synthesized by Pepscan (Netherlands) and used to interrogate the presence of antigen-specific T cells in HLA-A02:01+ GBM patients’ and healthy donors’ peripheral blood mononuclear cells (PBMCs). *In vitro* priming of PBMC with peptides of interest was performed as previously described [38, 39]. pMHC(HLA-A02:01)-tetramer loaded with peptides of interest was assembled as previously described [40]. 1 × 105 primed T cells were washed and resuspended in 100 μl PBS. 80 ng of pMHC-tetramer (in 2 μl) was added to the suspension and incubated at 37°C for 25 min. Cells were washed in PBS and further cell surface staining was performed at 4°C in PBS–2% FCS. Peptide-specific T cells identified by pMHC-tetramer staining were isolated by flow cytometry. Isolated T cells were further expanded and tested for functional reactivity against the respective peptide by co-culturing T cells and peptides at various concentrations, followed by immunostaining of TNF-a (clone MAb11, Biolegend) and CD107a (clone H4A3, Biolegend), and analysis by flow cytometry. Positive peptides were those where reactive T cells were confirmed to exist in GBM patients or healthy donors. To minimize variation in the current study, we additionally filtered for lengths 9- and 10-mer.

### Evaluating model performance against ‘GBM neoantigens’

Each model was executed as published and prediction scores were generated for each peptide in the ‘GBM’ dataset. ROC-AUC, PR-AUC, assessment metrics and confusion matrices were generated as described previously.

### Evaluating model performance against ‘TESLA consortium neoantigens’

The ‘TESLA’ dataset was downloaded from Wells *et al*. [3]. In their study, 608 predicted neoantigens were derived from six patients. These predicted neoantigens were tested for immunogenicity, and 37 of them were found to be immunogenic. Here, test peptides were filtered for lengths (9 and 10) and HLA alleles (see above) for which all models are applicable.

### Exploring differences in features associated with immunogenicity between pathogen and cancer datasets

The ‘TESLA’ dataset was acquired as above. All 608 peptides of lengths 8–14 were employed in this analysis. To curate the ‘pathogenic’ peptide dataset, we downloaded MHC class I peptides from the IEDB (accessed 17 February 2022). This dataset was then supplemented with pMHC from the *Repitope* package’s data repository, which was not found in the IEDB dataset. We retained only peptides of lengths 8–14 and with four-digit HLA resolution. Furthermore, we excluded any peptides from ‘antigen organisms’ containing the phrases ‘homo sapien’ or ‘cancer’. We excluded an additional 26 pMHCs that possessed only a single immunogenic observation but two or more non-immunogenic observations, which suggests that the positive assay could not be replicated. Otherwise, ‘contradictory’ observations (where a pMHC has been observed to be both immunogenic and non-immunogenic by different experiments) were considered immunogenic, adopting the approach described previously by Ogishi *et al*. [11]. We excluded any duplicated peptide-immunogenicity-MHC observations, arising from, e.g., multiple experimental assays yielding the same qualitative result. Lastly, only peptides predicted to bind their corresponding HLA allele (NetMHCpan 4.0 with a cutoff Rank threshold of 2.0) were retained for analysis. For analysis, NetMHCpan4.0 was used to predict binding affinities of peptides to corresponding HLAs. NetMHCstabpan [41] was used to predict binding stabilities. The ‘fraction of hydrophobicity’ was calculated as the fraction of a peptide’s residues that were hydrophobic. Hydrophobic residues were considered as ‘V’, ‘I’, ‘L’, ‘F’, ‘M’, ‘W’ and ‘C’ [3]. To measure the ‘fraction of hydrophobicity’ in TCR contact positions, we filtered only on peptides of lengths 9 and 10. Consistent with the approach of Koncz *et al*. [35], we defined TCR contact positions of 9-mers as positions 4 through 8, and such positions of 10-mers as positions 5 through 9. We then measured the fraction of each 5-mer that contained hydrophobic residues.

### HLA imbalance in model predictions for pathogenic epitopes: exploratory analysis

We gathered the prediction scores generated by testing models against the SARS-CoV-2 dataset after being tasked with predicting immunogenicity. If a peptide was observed to bind HLA-A02:01 in this data, it was labelled as ‘HLA-A02:01+’, while the remaining peptides were labelled ‘HLA-A02:01−’.

### HLA imbalance in model predictions for pathogenic epitopes: predicting HLA-A02:01+ peptides from SARS-CoV-2 peptide immunogenicity prediction scores

Prediction scores generated by each model after the SARS-CoV-2 experiment to evaluate performance in predicting immunogenicity were gathered. Instead of producing ROC curves against the ‘Immunogenicity’ column, however, ROC curves were produced against the binary classification of whether the peptide engages with HLA-A02:01 or not (i.e. HLA-A02:01+/−).

**Key Points**

- An unbiased systematic evaluation of several publicly available models that are commonly used to identify CD8+ T cell peptide targets for both pathogens and cancers is presented.
- For predicting immunogenic peptides from an emerging virus (SARS-CoV-2), none of the assessed models offered considerable improvements beyond HLA ligand prediction.
- While models could identify immunogenic cancer neoantigens, poor precision contributed to suboptimal overall performance in this setting.
- Cross-HLA variation in the distribution of immunogenic versus non-immunogenic peptides in training data appeared to confound predictions.
- Evidence indicated that different parameter thresholds may be needed for accurate prediction of immunogenic peptides in pathogens versus cancer.

## Supplementary Material

Supplementary_bbac141

BenchmarkingExtendedData_bbac141

Supplementary_TIFF_bbac141

## Data Availability

Key datasets generated or analyzed during this study are included in this published article and its supplementary information files. Datasets used for benchmarking can be found in [‘Extended Data’]. Code used to perform the analysis can be found on github: https://github.com/paulrbuckley/ImmunogenicityBenchmarkingOTB

## Author Biographies

**Paul R. Buckley** is a researcher at the Weatherall Institute of Molecular Medicine at the University of Oxford. His research involves the use of bioinformatics and/or mathematical modelling techniques to further understand immune responses in infectious disease, vaccines and cancer immunotherapies.

**Chloe H. Lee** is a PhD student at University of Oxford. Her research interest lies at Bioinformatics and Machine learning.

**Ruichong Ma** is an academic clinical lecturer in neurosurgery and a neurosurgery resident at the University of Oxford. His research interests are on glioma biology and therapeutics.

**Isaac Woodhouse** is a senior bioinformatician at the University of Oxford with an interest in bioinformatics and computational biology. Most recently he has specialised in proteogenomics and neoantigen prediction methods.

**Jeongmin Woo** is a postdoctoral researcher at the Weatherall Institute of Molecular Medicine at the University of Oxford.

**Vasily Tsvetkov** is the Chief Scientific Officer at ImmunoMind Inc., a US biotechnology company focused on the development of cell-based immunotherapies using single-cell technologies. He trained as a physician and graduated from Pirogov Russian National Research Medical University, majoring in medical biophysics. After graduation, he led projects on the computational prediction of immunogenicity and minor histocompatibility antigens leveraging statistical and machine learning models.

**Dmitrii Shcherbinin** is a postdoctoral researcher at the Immunosequencing Algorithms laboratory, Institute of Bioorganic Chemistry RAS, Moscow. His interests include protein structural analysis, molecular dynamics and modelling of peptide binding to the Major Histocompatibility Complex (MHC) molecules and studying interactions in T-cell receptor-peptide-MHC complexes.

**Agne Antanaviciute** is a postdoctoral researcher at the Weatherall Institute of Molecular Medicine at the University of Oxford. Her research interests are computational biology and immunology.

**Mikhail Shugay** is the head of Immunosequencing Algorithms laboratory at the Institute of Bioorganic Chemistry RAS, Moscow. His research interests include development of statistical methods and computational tools for immunogenetics data analysis. He has authored and co-authored MIXCR, VDJtools and MIGEC software and is the curator of the VDJdb database.

**Margarida Rei** is a postdoctoral fellow at the Ludwig Institute for Cancer Research at the University of Oxford.

**Alison Simmons** is Professor of Gastroenterology, Honorary Consultant Gastroenterologist and Director of the MRC Human Immunology Unit (MRC HIU) at the University of Oxford. She trained as a physician in London, Cambridge, and Oxford, specialising in gastroenterology. She undertook a DPhil and Clinician Scientist award with Professor Sir Andrew McMichael, FRS, investigating the mechanisms of HIV pathogenicity. Prof Simmons is an NIHR Senior Investigator, Wellcome Investigator and Fellow of the Academy of Medical Sciences.

**Hashem Koohy** is an Associate Professor of Systems immunology in the Radcliffe Department of Medicine and an Alan Turing Fellow at University of Oxford. His research focuses on providing deeper insights into the rules governing T cells recognition of pathogens.
