# Supplementary material for: Evaluating performance of existing computational models in predicting CD8+ T cell pathogenic epitopes and cancer neoantigens
Source: Brief Bioinform. 2022 Apr 25;23(3):bbac141. doi: 10.1093/bib/bbac141 (PMC9116217; doi:10.1093/bib/bbac141)
Supplement: Supplementary_bbac141 [file supplementary_bbac141.docx]

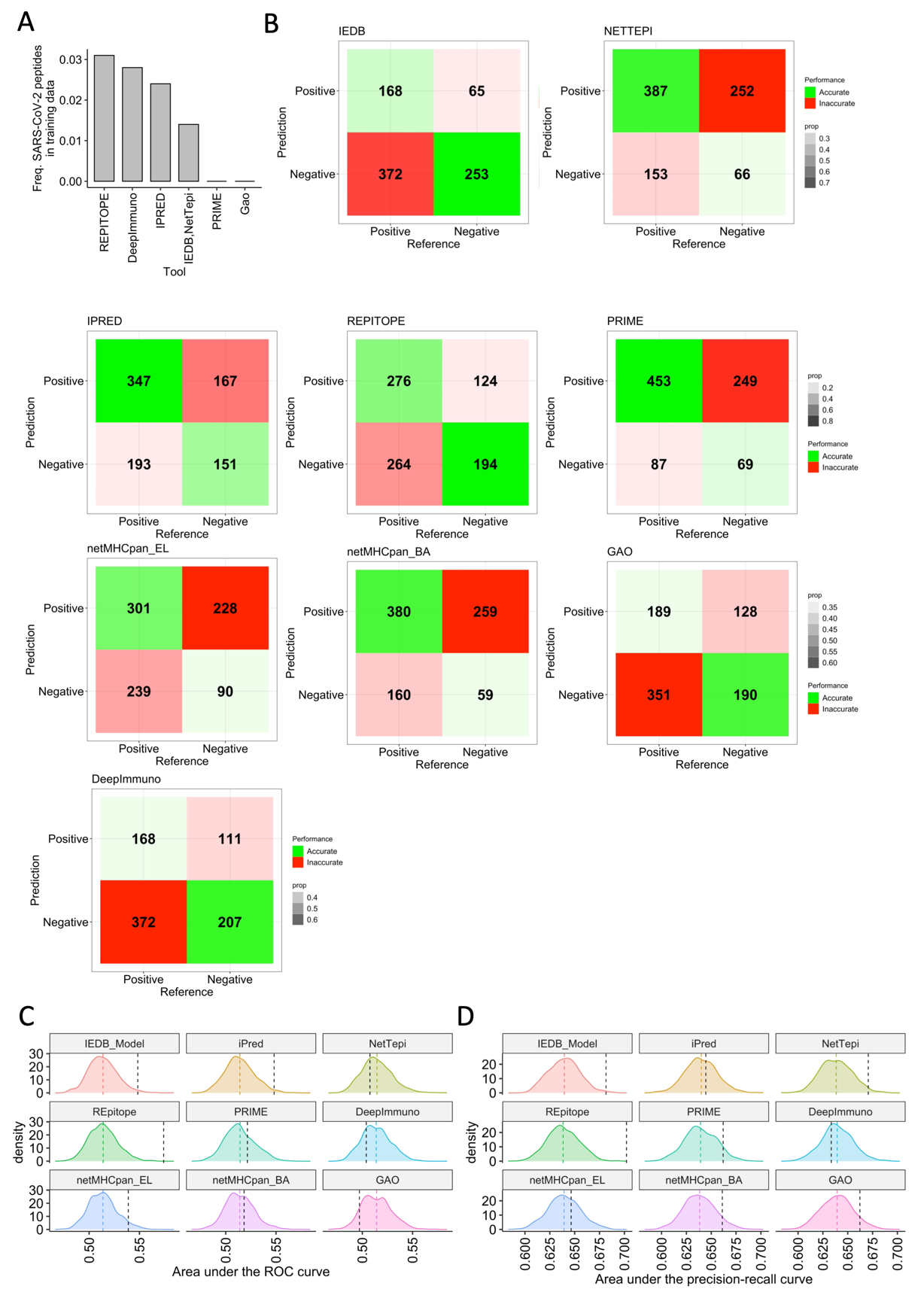


Supplementary Figure 1: A) Frequency of 858 SARS-CoV-2 peptides that exist in each model’s training data. As these models were published before the emergence of SARS-CoV-2, these peptides will represent homologs from e.g., MERS or SARS-CoV. B) Confusion matrices of models against SARS-CoV-2 dataset computed using youden index derived threshold on ROC-AUC data. C) Density plots showing model performance measured by ROC-AUC, relative to a bootstrapped random distribution. Black line shows the ‘true’ ROC-AUC measured from the performance evaluation. Coloured lines show the mean of the randomly generated distribution reflecting the background.


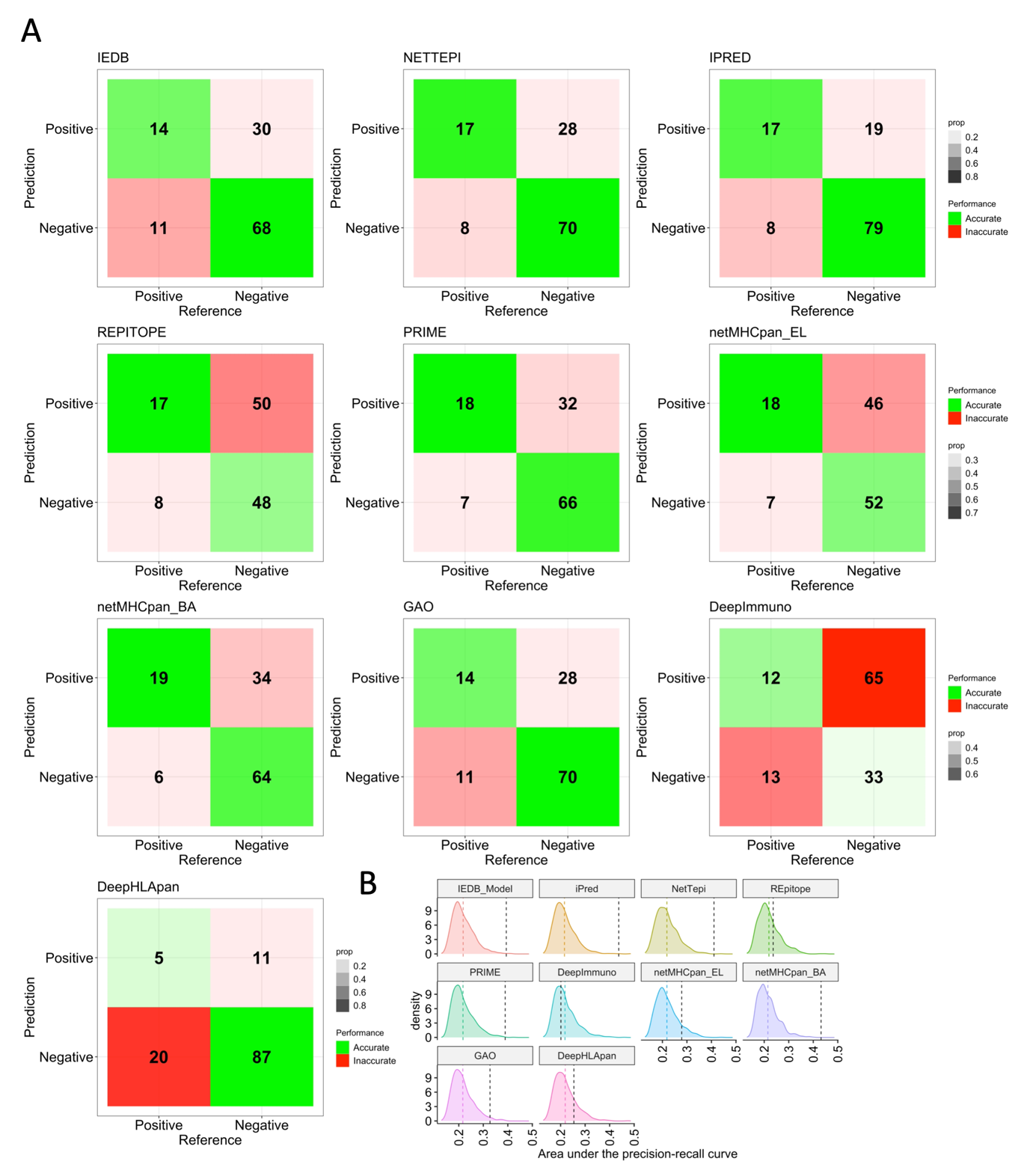


Supplementary Figure 2: A) Confusion matrices of models against unseen neo-peptides from glioblastoma patients. B) PR-AUCs of each model against the GBM dataset (black dashed line) contrasted with a random ‘bootstrap’ distribution.


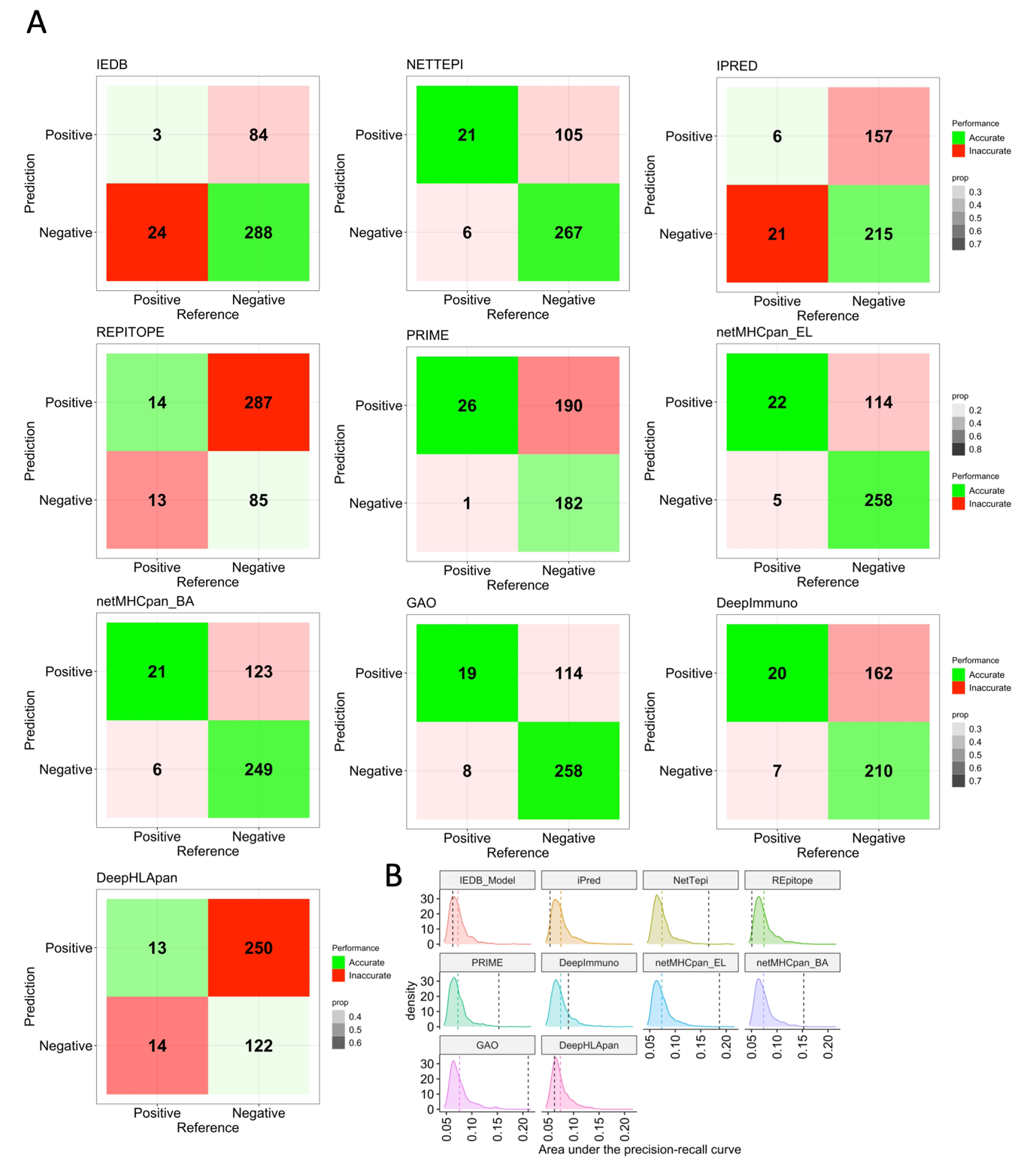


Supplementary Figure 3: A) Confusion matrices of models against unseen neo-peptides from the TESLA dataset. B) PR-AUCs of each model against the TESLA dataset (black dashed line) contrasted with a random ‘bootstrap’ distribution.


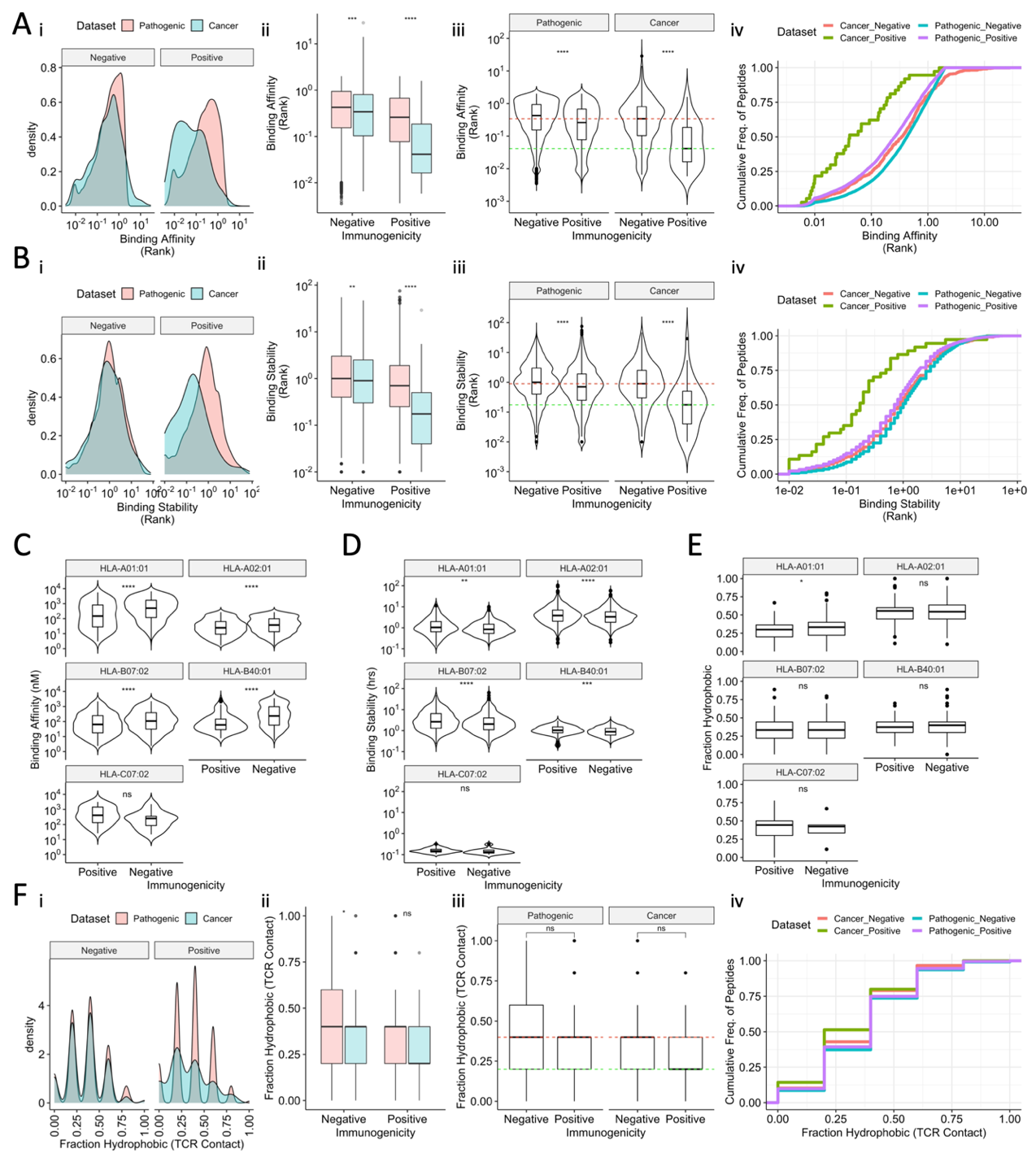


Supplementary Figure 4: Differences in discriminative features of immunogenicity: A-B) Analysis of the A) binding affinity rank scores, B) binding stability rank scores between pathogenic and cancer peptides. Density plots showing the distributions of binding affinity rank scores (A-i) or binding stability rank scores (B-i) for immunogenic and non-immunogenic peptides within pathogenic vs cancer datasets. ii) Boxplots comparing binding affinity rank scores (A-ii) or binding stability rank scores (B-ii) of immunogenic pathogen vs immunogenic cancer peptides, as well as non-immunogenic pathogen vs non-immunogenic cancer peptides. iii) Violin plots comparing the binding affinity rank scores (A-iii) or binding stability rank scores (B-iii) of pathogenic immunogenic vs. non-immunogenic peptides, as well as immunogenic vs. non-immunogenic cancer peptides. Green and red dashed lines show the median of the binding affinities for the immunogenic and non-immunogenic cancer peptides respectively. iv) Line plots showing the empirical cumulative distributions of binding affinity rank scores (A-iv) or binding stability rank scores (B-iv) grouped by whether the peptides are immunogenic or non-immunogenic for either cancer or pathogenic peptide datasets. C-D) Violin plots for five common HLAs, comparing the C) binding affinities (nM) and D) binding stabilities (hrs) between immunogenic and non-immunogenic pathogenic peptides. E) Boxplots for five common HLAs, comparing the fraction of the peptide that is hydrophobic, between immunogenic and non-immunogenic pathogenic peptides. F) Plots examining the ‘fraction of hydrophobicity’ in TCR contact positions of 9 and 10-mer peptides. F-i) Density plots showing the distributions of fraction of hydrophobic residues in TCR contact positions for immunogenic and non-immunogenic peptides within pathogenic vs. cancer datasets. F-ii) Boxplots of both immunogenic and non-immunogenic peptides, comparing the fraction of hydrophobic residues in TCR contact positions between pathogenic and cancer peptides. F-iii) Boxplots comparing the fraction of hydrophobicity in TCR contact positions between immunogenic vs. non-immunogenic pathogenic peptides, as well as immunogenic vs. non-immunogenic cancer peptides. Line plots showing the empirical cumulative distributions, grouped by whether the peptides are immunogenic or non-immunogenic and from cancer or from pathogens. Significance was assessed using Wilcoxon tests.


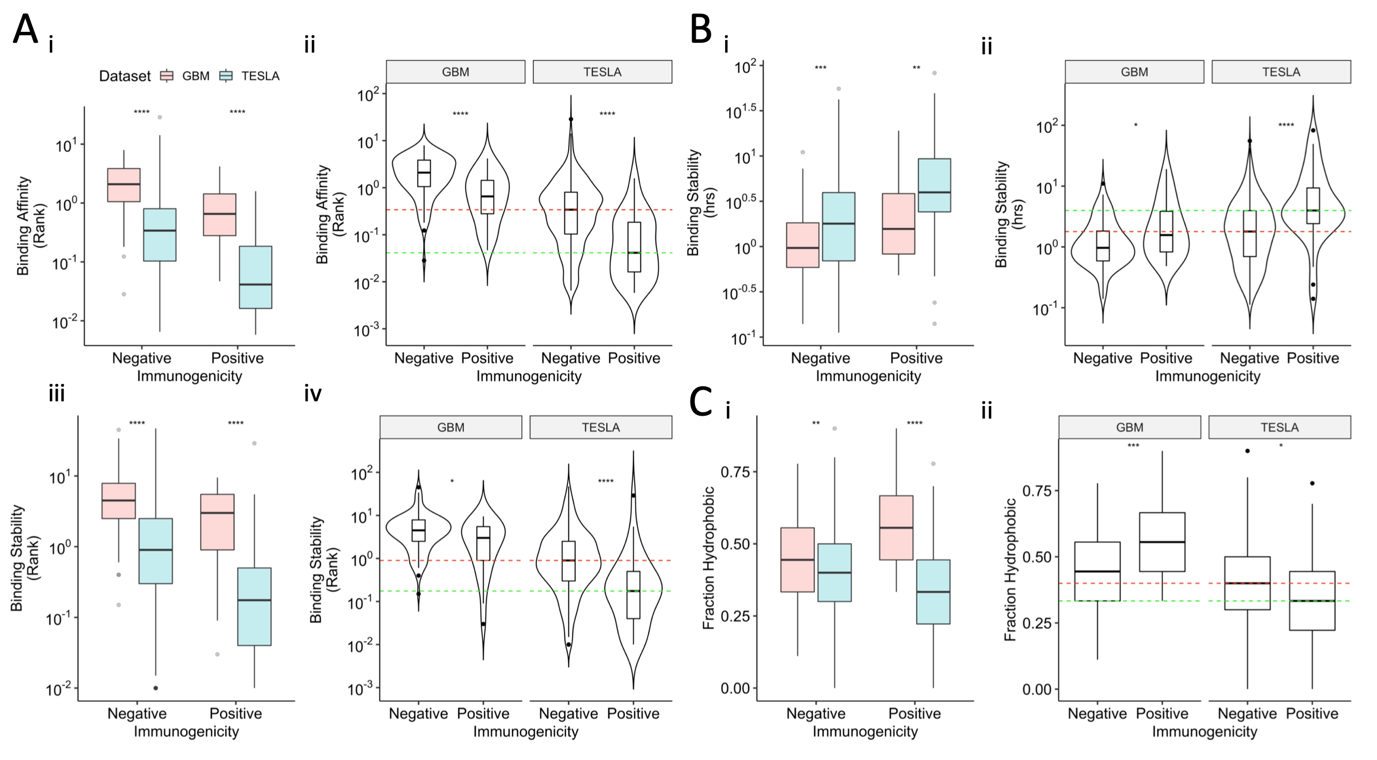


Supplementary Figure 5: Analysis comparing the presentation features associated with immunogenicity for two independent cancer datasets (GBM vs. TESLA). A) Comparing binding affinity rank scores of the GBM vs TESLA datasets. B) Comparing binding stability in hrs (B-i and B-ii) and rank scores (B-iii and B-iv) of the GBM vs TESLA peptides. C) Comparing fraction of hydrophobicity of the GBM vs. TESLA peptides. A-i, B-i, B-iii, C-i) boxplots comparing A-i) binding affinity rank scores, B-i) binding stabilities in hours, B-iii) binding stability rank scores, C-i) fraction of hydrophobicity of the GBM vs. TESLA peptides, grouped by whether the peptide is non-immunogenic or immunogenic. A-ii, B-ii, B-iv) Violin plots comparing A-ii) binding affinity rank scores, B-ii) binding stability in hours, B-iv) binding stability rank scores of GBM immunogenic vs. non-immunogenic peptides, as well as immunogenic vs. non-immunogenic TESLA peptides. Green and red dashed lines show the median of the respective measurement for the immunogenic and non-immunogenic TESLA peptides respectively. C-i) boxplots comparing the fraction of hydrophobicity of the GBM vs. TESLA peptides, grouped by whether the peptide is non-immunogenic or immunogenic. C-ii) Boxplots comparing the fraction of hydrophobicity of GBM immunogenic vs. non-immunogenic peptides, as well as immunogenic vs. non-immunogenic TESLA peptides. Green and red dashed lines show the median of the respective measurement for the immunogenic and non-immunogenic TESLA peptides respectively. Significance was assessed using Wilcoxon tests.


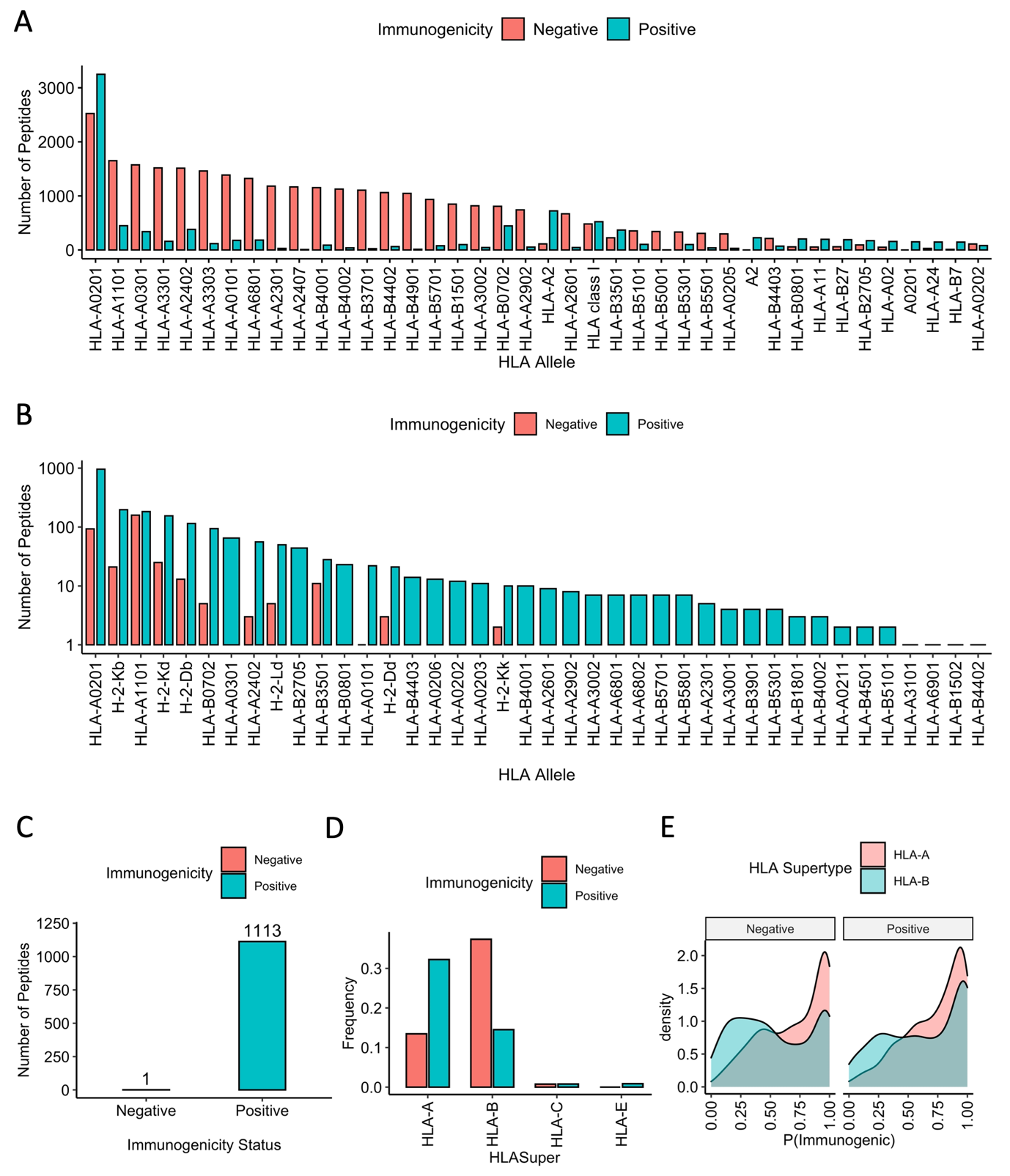


Supplementary Figure 6: A) Barplot showing the distribution of the 40 most common HLA allele groupings per immunogenicity status amongst Repitope’s MHCI human training dataset. B) Barplot showing the number of immunogenic and non-immunogenic peptides amongst the IEDB model training dataset. The y-axis is log10 scaled. C) Barplot showing the distribution of immunogenic and non-immunogenic peptides tested in the context of humans, for the IEDB model. D) Barplot showing the HLA supertype distribution for immunogenic and non-immunogenic peptides amongst the Chowell dataset used to train iPred. E) The distribution of immunogenicity scores per HLA-A or HLA-B supertypes for immunogenicity status, after training iPred canonically and asking the model to predict 2092 unseen peptides, with a balance of 523 peptides per HLA Supertype (HLA-A and HLA-B) and immunogenicity group.

S1 Table: Model metrics after performance evaluation against the SARS-CoV-2 dataset.

S2 Table: ROC-AUC bootstrap analysis metrics for each model against the SARS-CoV-2 dataset. ‘Bootstrap_mean’ column shows the mean of the randomly generated distribution. ‘Predicted’ shows the real ROC-AUCs achieved by each model.

S3 Table: PR-AUC bootstrap analysis metrics for each model against the SARS-CoV-2 dataset. ‘Bootstrap_mean’ column shows the mean of the randomly generated distribution. ‘Predicted’ shows the real PR-AUCs achieved by each model.

S5 Table: PR-AUC bootstrap analysis metrics for each model against the GBM dataset. ‘Bootstrap_mean’ column shows the mean of the randomly generated distribution. ‘Predicted’ shows the real PR-AUCs achieved by each model.


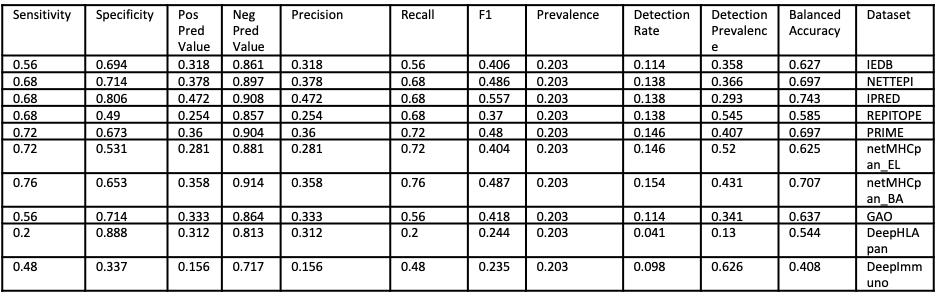


S4 Table: Model metrics after performance evaluation against the GBM dataset.


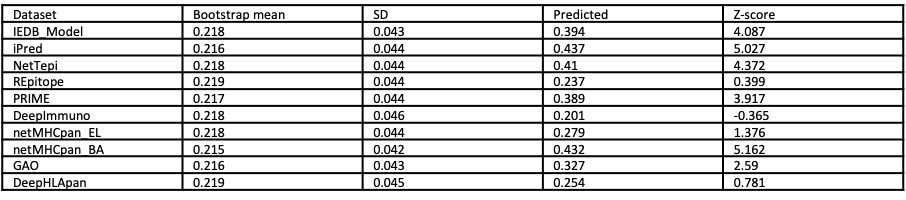


S5 Table: PR-AUC bootstrap analysis metrics for each model against the GBM dataset. ‘Bootstrap_mean’ column shows the mean of the randomly generated distribution. ‘Predicted’ shows the real PR-AUCs achieved by each model.


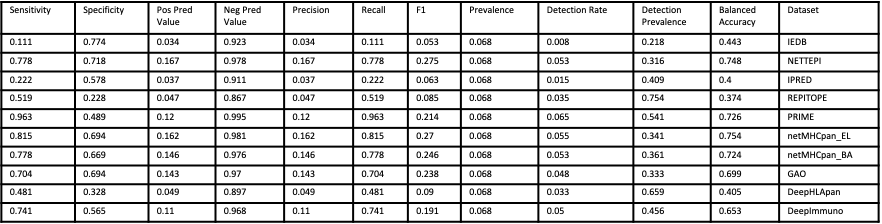


S6 Table: Model metrics after performance evaluation against the TESLA dataset.


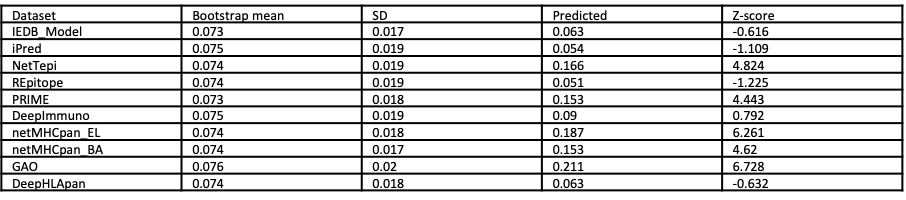


S7 Table: PR-AUC bootstrap analysis metrics for each model against the TESLA dataset. ‘Bootstrap_mean’ column shows the mean of the randomly generated distribution. ‘Predicted’ shows the real PR-AUCs achieved by each model.

| Reference | Model Description | Notes and Exclusion Justification |
| --- | --- | --- |
| Riley, T. P., Keller, G. L. J., Smith, A. R., Davancaze, L. M., Arbuiso, A. G., Devlin, J. R., & Baker, B. M. (2019). Structure based prediction of neoantigen immunogenicity. *Frontiers in Immunology*, *10*(AUG). https://doi.org/10.3389/fimmu.2019.02047 | Neural-network model trained on structural information within the context of TCRs and peptide binding. Aimed at predicting immunogenic neoantigens. | To our knowledge, the model is not publicly available. Additionally, this particular study focused om HLA-A*02:01-bound peptides while our work focused additionally on wider HLAs. |
| Jurtz, V. I., Jessen, L. E., Bentzen, A. K., Jespersen, M. C., Mahajan, S., Vita, R., Jensen, K. K., Marcatili, P., Hadrup, S. R., Peters, B., & Nielsen, M. (2018). NetTCR: sequence-based prediction of TCR binding to peptide-MHC complexes using convolutional neural networks. *BioRxiv*, 433706. https://doi.org/10.1101/433706 | NetTCR, which predicts binding probability between the CDR3 sequence of a TCR and an HLA-A02:01-peptide complex. NetTCR is a convolution neural network model, detecting patterns in input data with convolution filters. | The capacity of NetTCR to predict binding likelihood that an HLA-A*02:01-bound peptide-MHC complex may bind a *specific CDR3 sequence*, means that this model is out of the scope of the present study. |
| Tung, C. W., Ziehm, M., Kämper, A., Kohlbacher, O., & Ho, S. Y. (2011). POPISK: T-cell reactivity prediction using support vector machines and string kernels. *BMC Bioinformatics*, *12*(1), 446. https://doi.org/10.1186/1471-2105-12-446 | POPISK is a support vector machine approach and was the first reported immunogenicity model which constructed and utilise a large-scale dataset. | We were unable to access the webserver or locate a copy of the model. |
| Wang, G., Wan, H., Jian, X., Ouyang, J., Li, Y., Tan, X., Xu, Y., Zhao, Y., Lin, Y., & Xie, L. (2019). *INeo-Epp: T-cell HLA class I immunogenic or neoantigenic epitope prediction via random forest algorithm based on sequence related amino acid features*. https://doi.org/10.1101/697011 | *iNeoEpp* is a recent immunogenicity predictor inspired by the enrichment-score approach utilised in the IEDB model by Calis et al. Their model extracts sequence physicochemical information, eluted ligand rank, peptide entropy and ‘immunogenic frequency score’ which reflects amino acid distribution frequency differences between immunogenic and non-immunogenic peptides at TCR contact sites. | Only a webserver is currently available. Using this webserver, we found that some peptides amongst our test data were not predicted to bind HLA by iNeoEpp but were also given high ‘immunogenicity’ scores. We thus concluded that we wold not be able to fairly evaluate the performance of this model, as our datasets may include predicted non-binders. |

S8 Table: Models which were considered for analysis but excluded.

**Supplementary Text: Model Descriptions**

**IEDB Model**

The *IEDB model* captures sequence-based frequencies of specific amino acids to describe those which are more prevalent in immunogenic peptides compared with non-immunogenic peptides. Thus, ‘enrichment scores’ are combined with weights on amino acid positional importance for immunogenicity. The user is provided with a score between -1 and 1, to indicate likelihood of immunogenicity.

**NetTepi**

*NetTepi* is defined as the linear combination of binding affinity scores which are obtained by *NetMHCpan* algorithm, binding stability scores which are obtained by *NetMHCstab* and T cell propensity scores which are obtained similarly to the *IEDB model*:


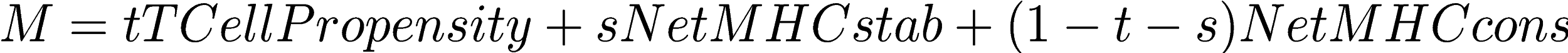


Developers optimized weights *t* and *s*, and defaults are supplied with the algorithm. *NetMHCstab* and *NetMHCcons* are called directly from *NetTepi* and individual scores for a given pMHC from these algorithms are produced. These are integrated as above with the T cell propensity predictions. To our knowledge, the only difference between the T cell propensity component of *NetTepi* and the *IEDB model*, is that only default masking (for HLA-A0201) to encode for positional importance is employed by *NetTepi*, whereas for the *IEDB model* there are different settings based on chosen HLA.

**iPred**

*iPred* is a Multinomial Gaussian Process classifier, utilising expectation-maximisation. As input *iPred* takes peptide sequence(s) and computes 10 Kidera Features averaged over all residues. The output of the model is a probability score reflecting the likelihood of T cell immunogenicity.

**Repitope**

*Repitope* is a framework which probes public TCRs to discriminate immunogenicity. *Repitope* computes physiochemical properties based on mimicking the thermodynamics between pMHC and public TCR interactions. This framework termed ‘TCR-peptide Contact Potential Profiling’ (CPP) focuses on the TCRβ CDR3 sequences, which are primarily involved in the interactions with peptides presented onto MHC. After a highly dimensional feature (~6000) computation occurs, feature selection can be performed and then a probabilistic estimate of immunogenicity is computed for each epitope. Models can be extrapolated to sequences for which it was not trained to make predictions.

*Repitope* can compute probabilistic estimates of the immunogenicity for an original epitope and for all single amino acid variants, as well as the largest difference of immunogenicity between variants to explore escape potential. *Repitope* also provides utility to predict CD4+ T cell immunogenicity for class II MHC.

**PRIME**

PRIME predicts immunogenic epitopes through capturing and deconvoluting molecular properties of antigen-presentation and TCR recognition propensity. The authors were able to achieve this deconvolution by a ‘careful annotation and analysis of epitope residues with minimal impact on binding to HLA-I molecules’. PRIME is efficient and runs faster than many HLA-I ligand predictors. For the HLA binding component of the prediction, PRIME uses MixMHCpred.

**DeepImmuno**

*DeepImmuno-CNN* is a convolution-neural-network approach which predicts immunogenicity of pMHC complexes. A beta-binomial probabilistic model is fit to the training dataset to generate an immunogenicity score. Their immunogenicity score weights each pMHC complex based on the strength of available experimental evidence in the training dataset. A principal component analysis of 566 amino acid physicochemical properties encodes each amino acid sequence.

**Gao**

A ‘physics-based’ learning model, aimed at predicting CD8+ T cell epitopes for a particular HLA genotype. The model is trained and validated on immunodominance hierarchy of CD8+ T cell epitope targets from HIV. They define a ‘CTL response metric’ which is the product of three terms. The terms capture 1) a measure of the probability that a peptide of interest is presented by a given HLA, 2) the sequence similarity between the peptide of interest to pathogen-derived peptides from the IEDB, and the sequence similarity of this peptide to peptides derived from the human proteome.
