# Supplementary figures and images for: Evaluating performance of existing computational models in predicting CD8+ T cell pathogenic epitopes and cancer neoantigens
Source: Brief Bioinform. 2022 Apr 25;23(3):bbac141. doi: 10.1093/bib/bbac141 (PMC9116217; doi:10.1093/bib/bbac141)

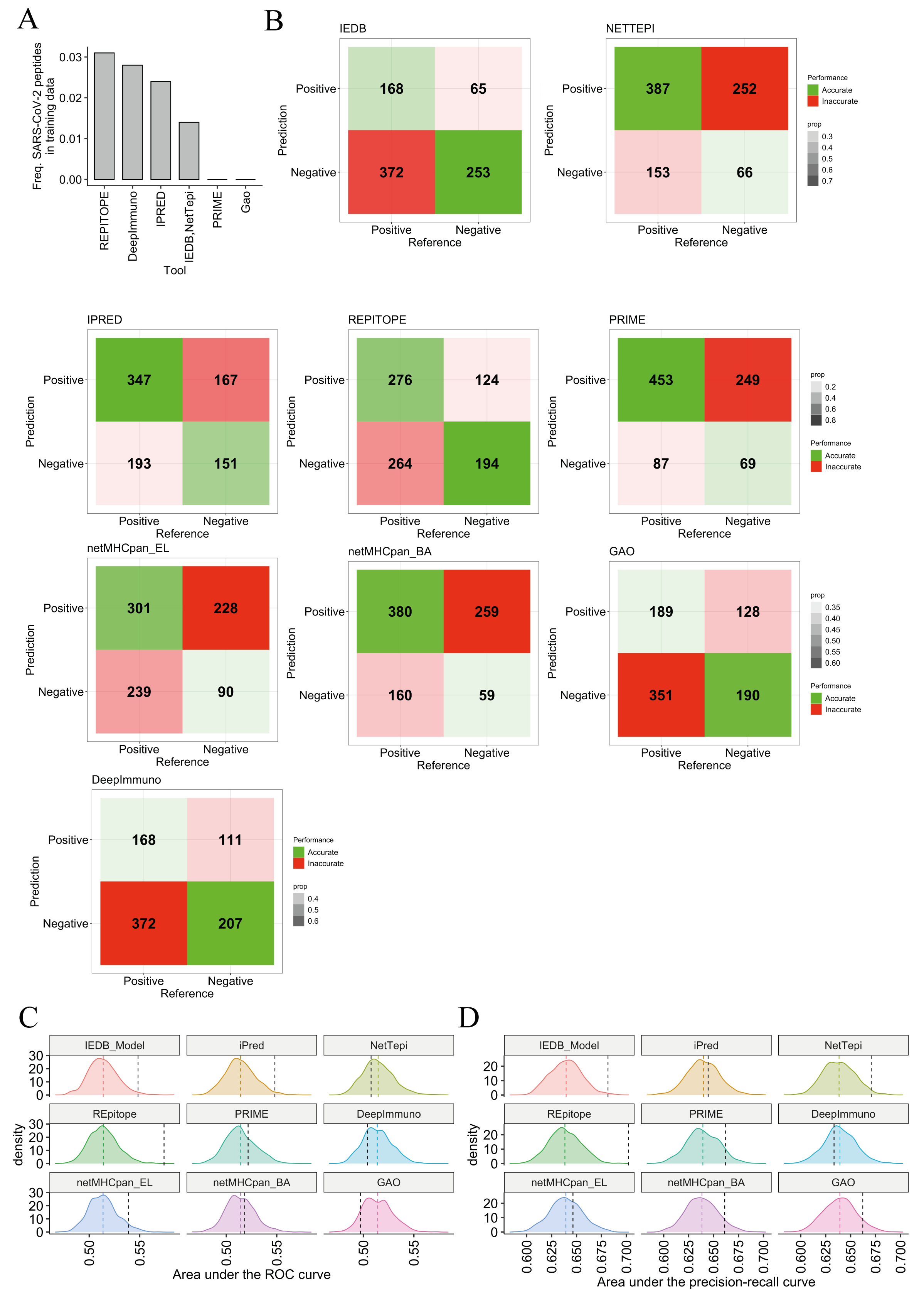

Supplement: Supplementary_TIFF_bbac141 [file supplementary_tiff_bbac141.zip › Supplementary_TIFF/S1.tif]

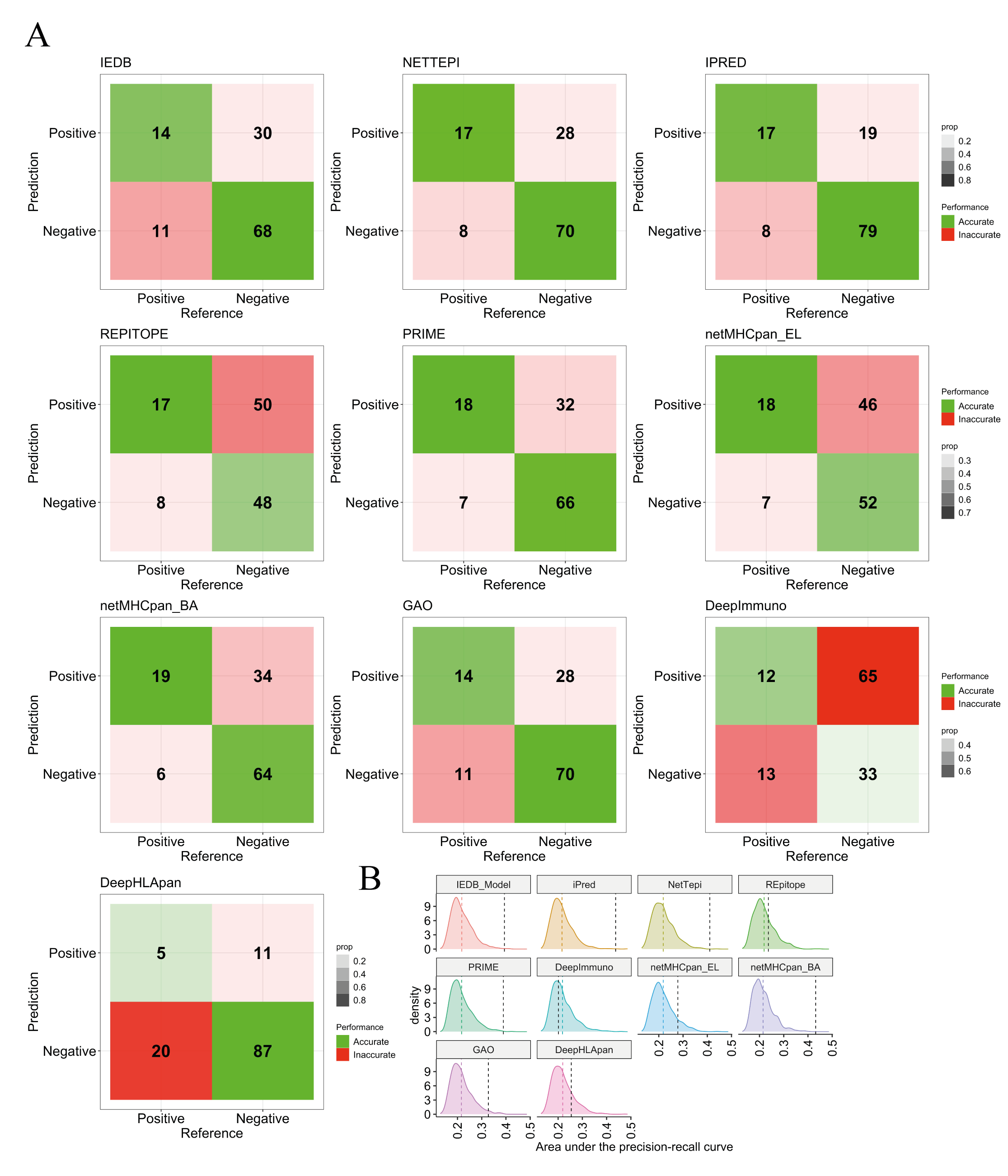

Supplement: Supplementary_TIFF_bbac141 [file supplementary_tiff_bbac141.zip › Supplementary_TIFF/S2.tif]

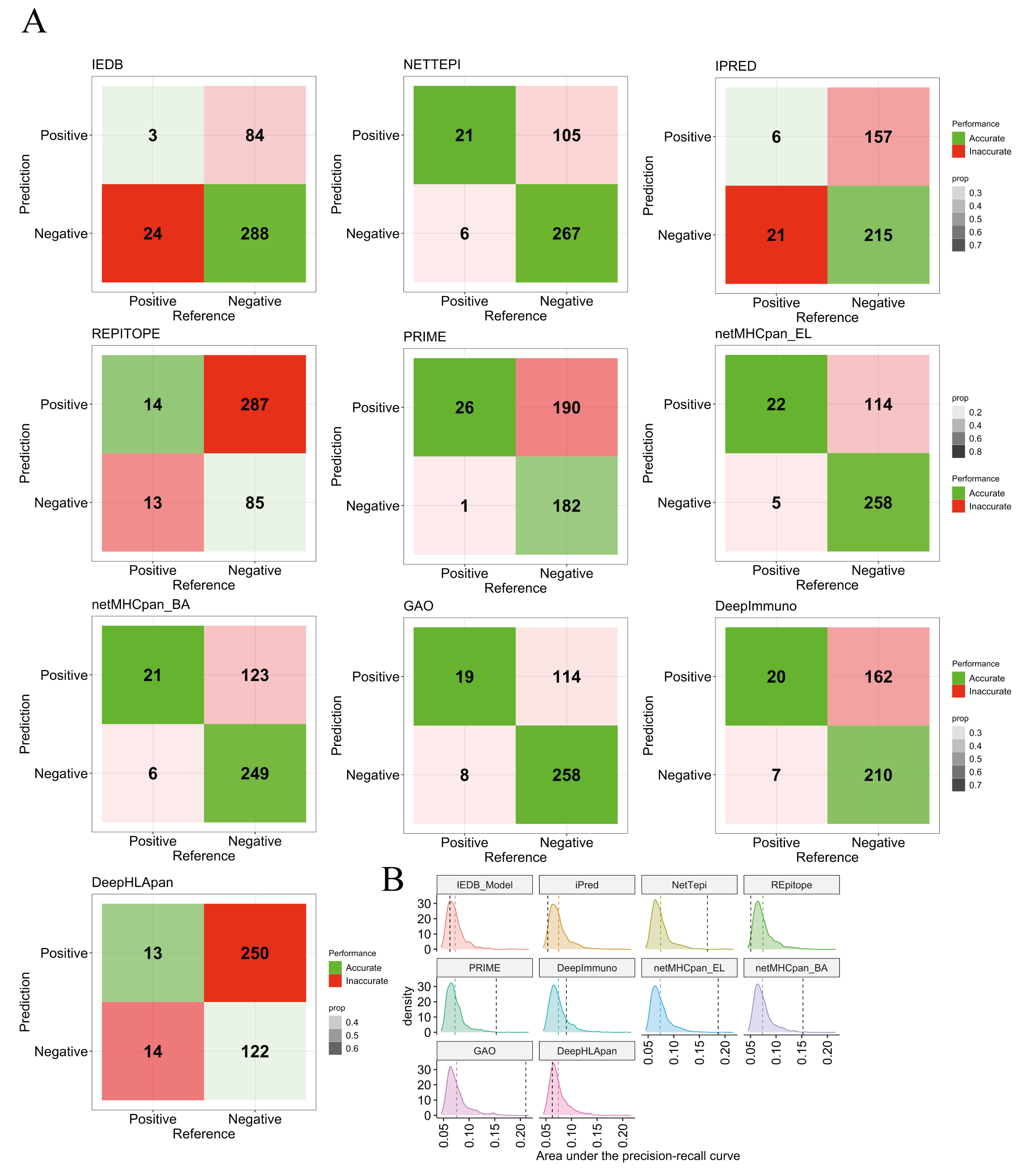

Supplement: Supplementary_TIFF_bbac141 [file supplementary_tiff_bbac141.zip › Supplementary_TIFF/S3.tif]

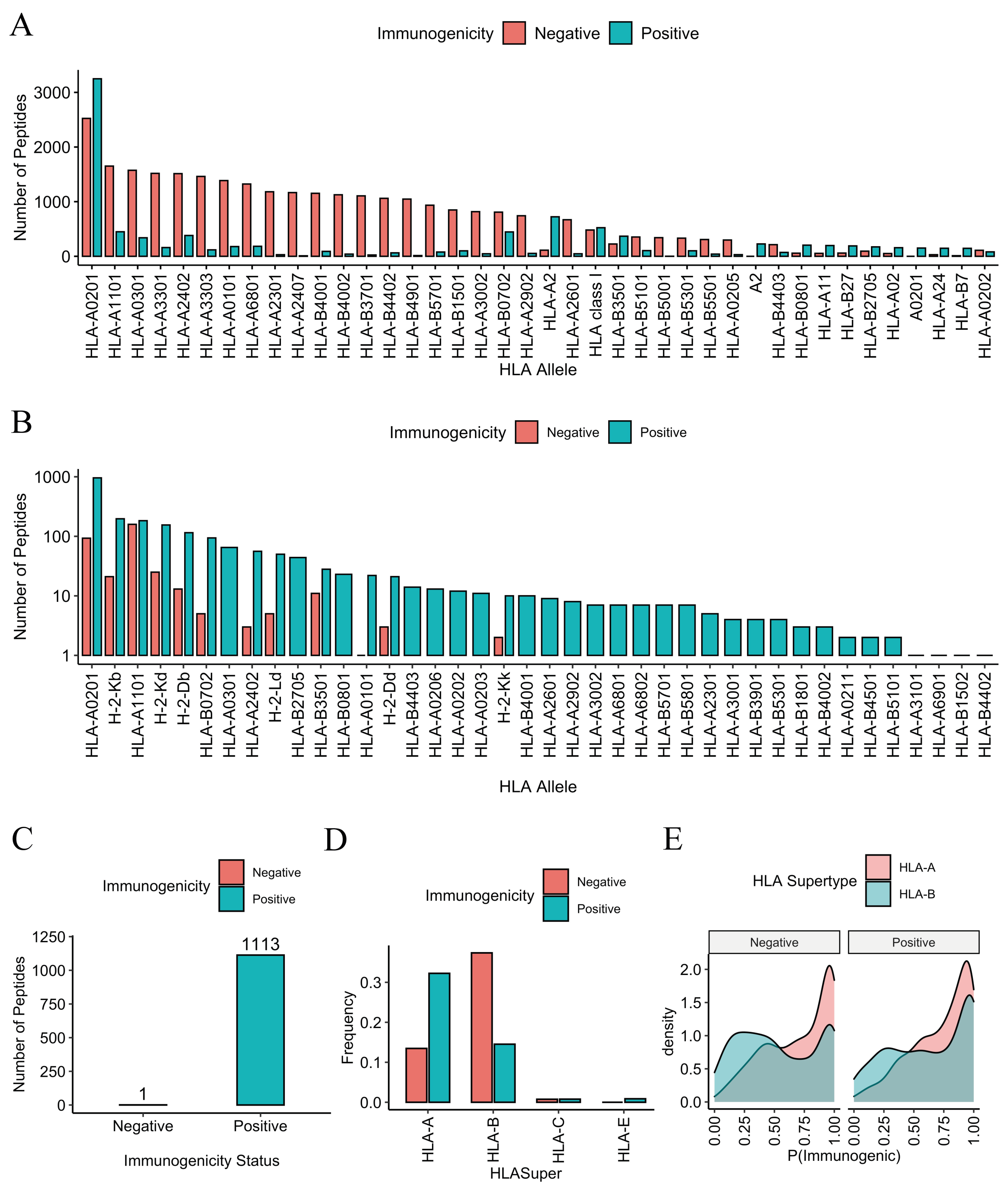

Supplement: Supplementary_TIFF_bbac141 [file supplementary_tiff_bbac141.zip › Supplementary_TIFF/S6.tif]

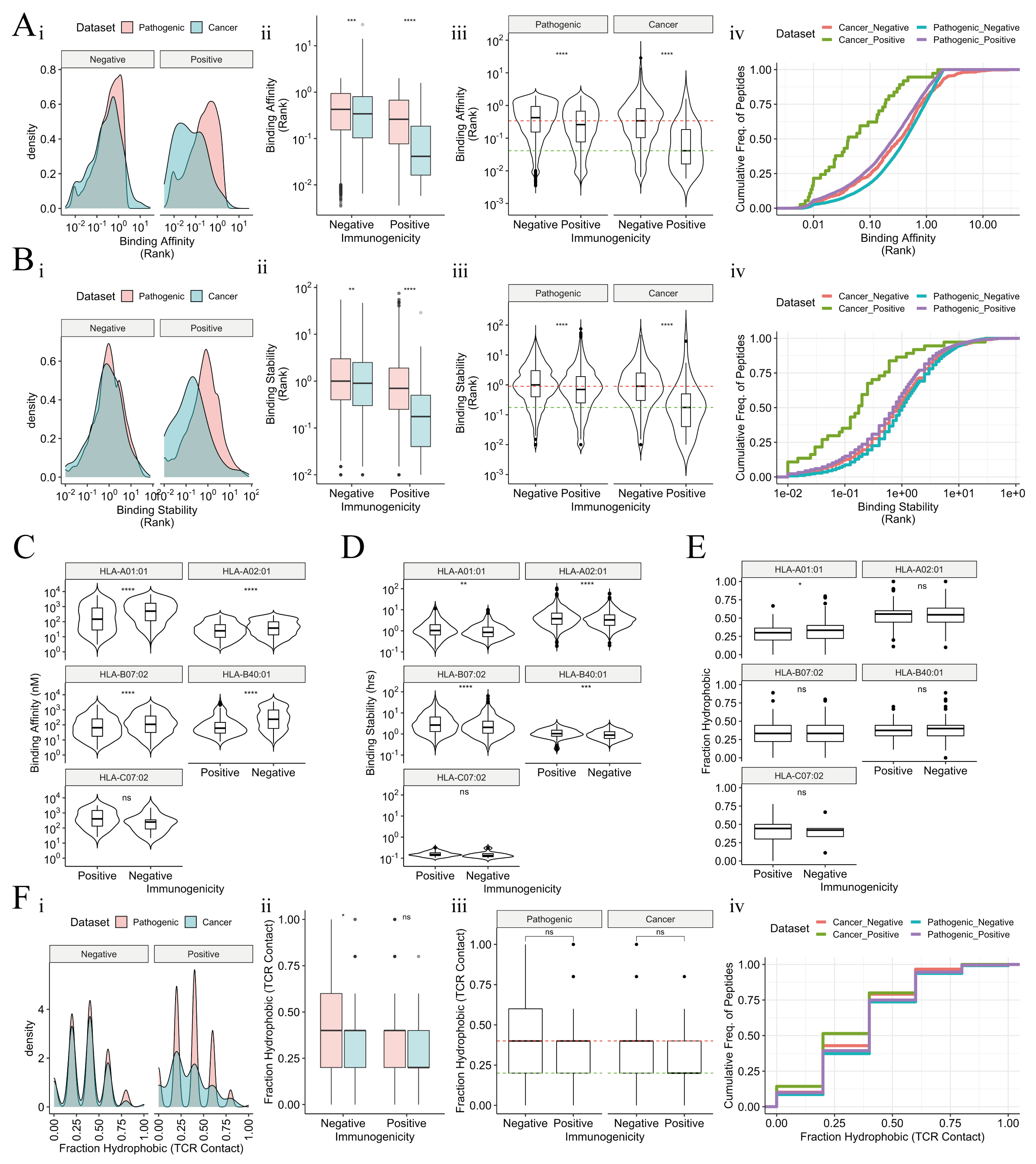

Supplement: Supplementary_TIFF_bbac141 [file supplementary_tiff_bbac141.zip › Supplementary_TIFF/S4.tif]

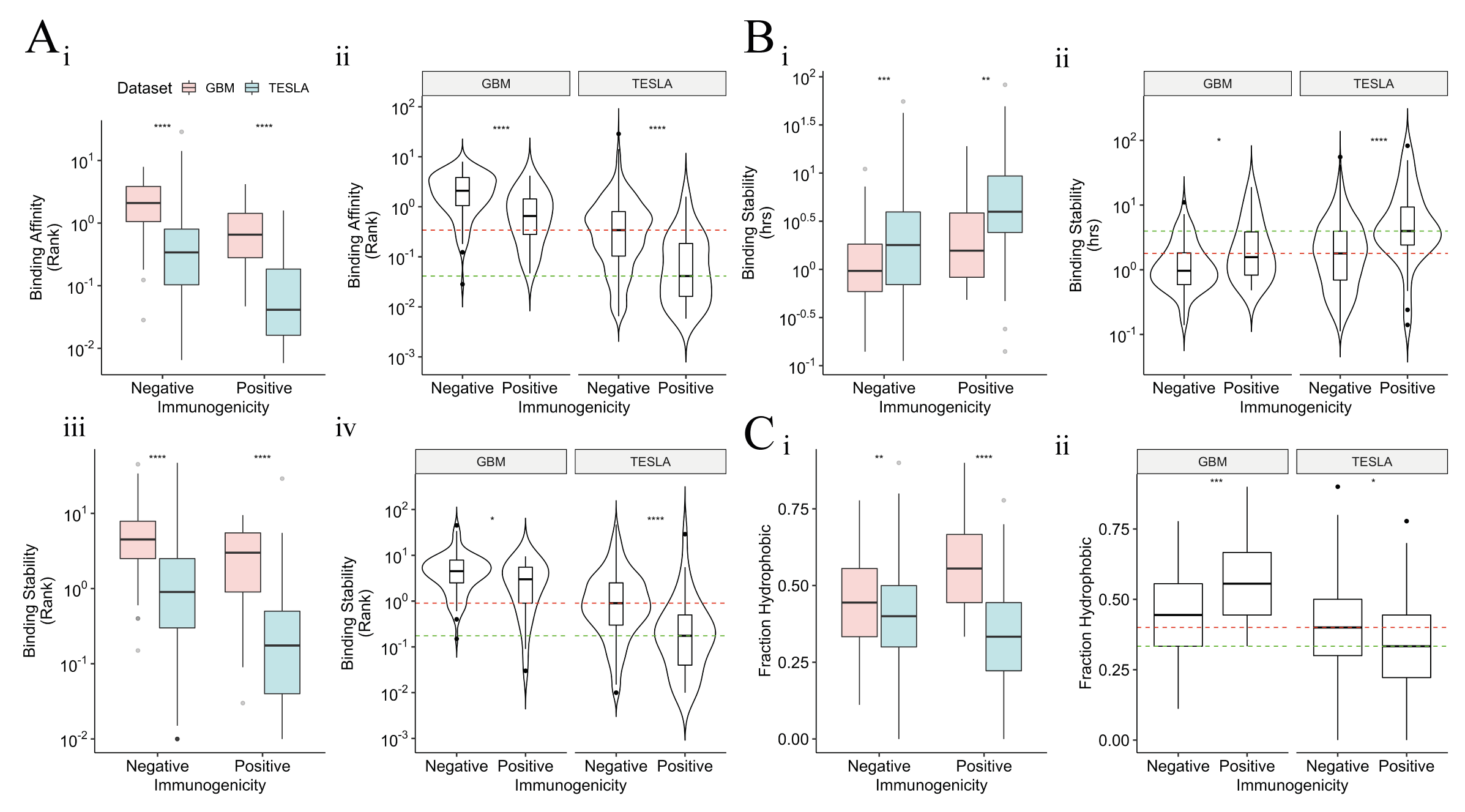

Supplement: Supplementary_TIFF_bbac141 [file supplementary_tiff_bbac141.zip › Supplementary_TIFF/S5.tif]
